# Determining the extent and frequency of on-site monitoring: a bayesian risk-based approach

**DOI:** 10.1186/s12874-024-02261-y

**Published:** 2024-06-28

**Authors:** Longshen Xie, Lin Liu, Shein-Chung Chow, Hui Lu

**Affiliations:** 1 Department of Bioinformatics and Biostatistics, School of Life Sciences and Biotechnology, SJTU-Yale Joint Center for Biostatistics and Data Science, Shanghai Jiao Tong University, No 800, Dongchuan Road, Minhang, 200240 Shanghai China; 2 Institute of Natural Sciences, MOE-LSC, School of Mathematical Sciences, CMA-Shanghai, SJTU-Yale Joint Center for Biostatistics and Data Science, Shanghai Jiao Tong University, No 800, Dongchuan Road, Minhang, 200240 Shanghai China; 3 Department of Biostatistics and Bioinformatics, Duke University School of Medicine, 2424 Erwin Road, Suite 11037, Durham, 27705 North Carolina USA

**Keywords:** Risk-based monitoring, Key risk indicators, Optimal risk boundary, Trial quality, Cost-effectiveness

## Abstract

**Background:**

On-site monitoring is a crucial component of quality control in clinical trials. However, many cast doubt on its cost-effectiveness due to various issues, such as a lack of monitoring focus that could assist in prioritizing limited resources during a site visit. Consequently, an increasing number of trial sponsors are implementing a hybrid monitoring strategy that combines on-site monitoring with centralised monitoring. One of the primary objectives of centralised monitoring, as stated in the clinical trial guidelines, is to guide and adjust the extent and frequency of on-site monitoring. Quality tolerance limits (QTLs) introduced in ICH E6(R2) and thresholds proposed by TransCelerate Biopharma are two existing approaches for achieving this objective at the trial- and site-levels, respectively. The funnel plot, as another threshold-based site-level method, overcomes the limitation of TransCelerate’s method by adjusting thresholds flexibly based on site sizes. Nonetheless, both methods do not transparently explain the reason for choosing the thresholds that they used or whether their choices are optimal in any certain sense. Additionally, related Bayesian monitoring methods are also lacking.

**Methods:**

We propose a simple, transparent, and user-friendly Bayesian-based risk boundary for determining the extent and frequency of on-site monitoring both at the trial- and site-levels. We developed a four-step approach, including: 1) establishing risk levels for key risk indicators (KRIs) along with their corresponding monitoring actions and estimates; 2) calculating the optimal risk boundaries; 3) comparing the outcomes of KRIs against the optimal risk boundaries; and 4) providing recommendations based on the comparison results. Our method can be used to identify the optimal risk boundaries within an established risk level range and is applicable to continuous, discrete, and time-to-event endpoints.

**Results:**

We evaluate the performance of the proposed risk boundaries via simulations that mimic various realistic clinical trial scenarios. The performance of the proposed risk boundaries is compared against the funnel plot using real clinical trial data. The results demonstrate the applicability and flexibility of the proposed method for clinical trial monitoring. Moreover, we identify key factors that affect the optimality and performance of the proposed risk boundaries, respectively.

**Conclusion:**

Given the aforementioned advantages of the proposed risk boundaries, we expect that they will benefit the clinical trial community at large, in particular in the realm of risk-based monitoring.

**Supplementary Information:**

The online version contains supplementary material available at 10.1186/s12874-024-02261-y.

## Background

For clinical trials that evaluate new medical treatments or devices, sponsors are responsible for monitoring the underlying study to ensure (i) that the enrolled subjects’ rights and safety are protected and (ii) that the protocol of the trial is strictly followed so that the collected data is of high quality and integrity. Baigent et al. [1] classified clinical-trial monitoring into three main categories: oversight by the trial committee, on-site monitoring, and centralised monitoring. These monitoring strategies often work in concert to ensure the compliance and validity of clinical trials. Oversight by trial committees involves a group of experts, which may include sponsors or independent third-party personnel such as the independent data monitoring committee (IDMC). Their primary objective is to review the safety, efficacy, and study design throughout the trial. On-site monitoring refers to the assessment conducted by sponsors or their representatives at clinical sites to verify the authenticity and integrity of trial records, ensure adherence to the study design, and assess investigators’ familiarity with the protocol. In contrast to on-site monitoring, centralised monitoring examines the trial quality remotely by analysing the accumulated data.

Centralised monitoring has garnered tremendous attention in recent years mainly due to the following reasons. First, several studies demonstrate that 100% source data verification (SDV), even incurring high cost, is insufficient for achieving satisfactory monitoring outcomes. For example, a recent retrospective study [14] analysing 1168 Phase I-IV trials conducted by 53 sponsors revealed that, in median, SDV only corrected 32.0% of all corrections in the case report form. Other issues are identified through auto-queries or other data-cleaning methods, such as medical and biostatistics reviews. Similarly, another study [15] found that SDV only contributed 7.8% of all queries. These findings highlight the limited role of untargeted on-site monitoring and indicate the need for sponsors to prioritise more important aspects. Second, the approval rate for new drugs from Phase I to a successful new drug application (NDA) is typically less than 10% [6]. Meanwhile, despite the increasing costs of research and development (R &D), the return rate on R &D investments has decreased from 10.1% in 2010 to 1.8% in 2019 [7]. A report [4] showed that on-site monitoring in large global clinical trials may cost up to 30% of the total budget. In other words, sponsors have the potential to achieve substantial cost savings if there are alternatives to on-site monitoring. Third, regulatory agencies from various countries and regions have published guidelines concerning centralised monitoring [5, 8, 17, 18]. Unsurprisingly, these guidelines advocate for the use of centralised monitoring and emphasise its importance. For example, the FDA [17] advises sponsors to develop a monitoring plan that addresses the specific risks related to ethics and data integrity in their trial. The benefits of centralised monitoring are also illustrated in the ICH E6(R2) guideline [9]. Lastly, considering the budget limitations faced by investigator-led clinical trials, where extensive on-site monitoring may not be feasible, centralised monitoring becomes particularly vital [3].

As an essential tool for implementing centralised monitoring, the concept of risk-based monitoring (RBM) was originally defined in an EMA reflection paper [8] as “a systematic process put in place to identify, assess, control, communicate, and review the risks associated with the clinical trial during its lifecycle”. Currently, there are two approaches for RBM based on trial- and site-levels, respectively. The ICH E6(R2) guideline [9] introduces a seven-step risk-based approach and suggests the establishment of quality tolerance limits (QTLs) to identify trial- or system-level critical data or processes, such as the incidence proportion of adverse events of special interest (AESI), which can impact the safety of clinical trial subjects or the reliability of trial results [2]. On the other hand, TransCelerate proposed the concept of “thresholds” primarily for assessing risks related to critical data or processes at the site-, country-, or protocol-level [15], such as discontinuation rate. For critical data or processes at the trial- and site-levels, there can be instances where their meanings overlap. For example, the rate of major protocol deviations can serve as a parameter at the trial-level or as a risk indicator at the site-level. The difference between them lies in the use of different criteria, such as QTLs or thresholds, for risk assessment. In addition, TransCelerate [16] used the terms “parameters” and “risk indicators” to represent critical data or processes at the trial- and site-levels, respectively.

Using QTLs or thresholds, sponsors can guide the extent and frequency of on-site monitoring and take corresponding monitoring actions by assessing the risk levels of a trial or site, respectively. For example, we use the discontinuation rate as a key indicator to assess the site’s risk level. We estiablish three risk levels, whose monitoring actions correspond to that on-site monitoring is required (high-risk), recommended (medium-risk), or unnecessary (low-risk), respectively. Assuming the thresholds for determining different risk levels are set at 10% and 30%, where the discontinuation rate below or equal to 10% indicates a low-risk level, above 30% indicates a high-risk level, and between the two indicates an medium-risk level. For example, if a site’s discontinuation rate is 35%, it would be assessed as a high-risk site requiring on-site monitoring.

Despite the potential merits listed in last paragraph, the aforementioned methods have two main limitations. First, the specific reason for defining QTLs or thresholds is typically not explicitly described. For instance, TransCelerate [15] only provided some rules-of-thumb, such as defining the discontinuation rate for high-risk levels as 30% more or less than the expected rate or a minimum of 4 subjects discontinued. Zink et al. [21] also pointed out that TransCelerate [15] did not even clearly explain whether it was relative or absolute change that was considered for the thresholds. Second, in comparison to larger sites, sites with a small number of subjects may have a high incidence proportion for a binary risk indicator. To address this issue, Zink et al. [21] advocated using *funnel plots* to identify risk indicators with outliers, where the thresholds were determined based on the lower and upper ends of a two-sided nominal (conservative) 95% confidence interval. The thresholds in the funnel plot increase as the sample size decreases. Furthermore, Zink et al. argued that TransCelerate’s method requires defining different thresholds based on various risk indicators, which can be very time-consuming. Consequently, the thresholds in the funnel plot are usually used only to differentiate between therapeutic areas or populations. However, they only considered discrete endpoints and did not further investigate continuous or time-to-event endpoints. In real trials, there are risk indicators related to these types of endpoints, as evidenced by the examples in TransCelerate’s position paper [15].

Due to these limitations, the primary objective of this paper is to find a widely applicable and explicit risk-based monitoring method that can assist in guiding and adjusting the extent and frequency of on-site monitoring. To avoid confusion with the terminology used in the ICH E6(R2) guideline and TransCelerate’s position paper [15], we introduce the term “key risk indicators” (KRIs) to represent critical data or processes at both the trial- and site-levels rather than using parameters or risk indicators, while using the term “boundaries” as a substitute for QTLs or thresholds. In contrast to the aforementioned methods, we propose the implementation of a Bayesian interval design to minimise the decision error rate based on different risk levels for critical data or processes [10, 20]. The decision error refers to a failure to accurately assess the risk level of a KRI through the proposed risk boundary. The steps involved in our method are as follows:

1. Establishing risk levels for KRIs along with their corresponding monitoring actions and estimates.
2. Calculating the optimal risk boundaries based on the risk level estimates.
3. Determining the risk level to which a trial or site belongs by comparing its KRIs’ outcomes against the optimal risk boundaries.
4. Providing recommendations and suggestions for on-site monitoring based on the comparison results.

Here, we need to clarify the difference between risk level estimates and risk boundaries. The former refers to the estimate of a KRI for different risk levels in a trial or site. For example, the incidence proportions of AESI for high-risk and low-risk sites are estimated to be 20% and 5%, respectively. Risk boundaries are criteria used to determine which risk level a site belongs to when its incidence proportion of AESI falls between 20% and 5%. The proposed method can determine the risk level of a trial or site based on one or multiple KRIs. It is applicable to a wide range of clinical trials, from Phase I to Phase IV, single or multicenter, at both the trial- and site-levels, although in this paper we primarily focus on the latter.

The rest of this paper is structured as follows. In Method section, we introduce the statistical theory that forms the basis of the proposed method and illustrate its advancements over existing methods. We then apply the general methodology to find KRIs for various common distributions, including Poisson, binomial, exponential, and normal distributions. Subsequently in Simulation, Results, and Example sections, we evaluate the performance of the proposed method in simulations and a real case study. We discuss the influential factors for the proposed method and provide additional considerations in Discussion section.

## Method

### Notations for the observed KRIs’ outcomes

Let $\textbf{X} {:=} (X_{jk})$ be a $J\times K$ KRI matrix, where ${X}_{jk}$ is an independent random variable, and its value ${x}_{ijk}$ denotes the observed outcome of the ${j}^{\text{th}}$ KRI for the ${i}^{\text{th}}$ subject at the ${k}^{\text{th}}$ trial or site, for $i = 1, \dots , n_{jk}$, $j = 1, \dots , J$, and $k = 1, \dots , K$. $n_{jk}$ counts the number of subjects with the $j^{\text{th}}$ KRI recorded at the $k^{\text{th}}$ trial or site. Typically, *K* equals 1 at the trial-level monitoring.

If the occurrence of the $j^{\text{th}}$ KRI can only be recorded once during the course of the trial, such as early withdrawal, then $x_{ijk}=1$ if it occurs, and otherwise $x_{ijk} = 0$. In this case, we can assume that $X_{jk}$ follows a binomial distribution $\text {Bin}(P_{jk},n_{jk})$ (with the total Bernoulli trial number equal to 1), where $P_{jk}$ denotes the true incidence proportion of the $j^{\text{th}}$ KRI at the $k^{\text{th}}$ trial or site. If the event of the $j^{\text{th}}$ KRI, such as the number of AESIs, may occur one or multiple times for the same subject, then $X_{jk}$ can be assumed to follow a Poisson distribution $\text {Poisson}(\Lambda _{jk})$, where $\Lambda _{jk}$ denotes the true average number of events or risk rate of the $j^{\text{th}}$ KRI per subject at the $k^{\text{th}}$ trial or site in a unit time. If $X_{jk}$ represents a time-to-event KRI, such as the duration from the first dose to the occurrence of an AESI, one can assume that $X_{jk}$ follows an exponential distribution $\text {Exp}(\Lambda _{jk})$. Finally, if the ${j}^{\text{th}}$ KRI is a continuous endpoint, $X_{jk}$ can be assumed to follow a normal distribution $\mathcal {N}( M _{jk},\Sigma _{jk}^2)$, where $M _{jk}$ and $\Sigma _{jk}$ denote the mean and standard error of the $j^{\text{th}}$ KRI at the $k^{\text{th}}$ trial or site.

### Establishing risk levels for KRIs along with their corresponding monitoring actions and estimates

In our method, the risk assessment of a trial or site is based on KRIs. Thus, we need to first establish *G* risk levels and corresponding monitoring actions for KRIs. It is generally recommended to establish two ($G=2$) or three ($G=3$) risk levels that align with those of QTLs or TransCelerate’s thresholds. For example, in TransCelerate’s position paper, they defined high-, medium-, and low-risk levels and provided the corresponding monitoring actions, such as on-site monitoring for high-risk sites and no such action for low-risk sites. In real clinical trials, the number of risk levels can be tailored to specific requirements.

Next, we need to define estimates of KRIs at different risk levels and assume they follow different distributions based on KRI types. For instance, we assume the estimate of the $j^{\text{th}}$ binary KRI at the $g^{\text{th}}$ risk level follows a binomial distribution $\text {Bin}(p_{jg},n_{jk})$, where $p_{jg}$ denotes the estimated incidence proportion of the $j^{\text{th}}$ KRI at the $g^{\text{th}}$ risk level and $g={1,2,\dots ,G}$. Without loss of generality, we order the risk levels as follows: $p_{j1}>p_{j2}>\dots >p_{jG}$, which means $g=1$ represents the highest risk level, while $g=G$ corresponds to the lowest risk level. For example, the estimated high- and medium-risk incidence proportions of the $j^{\text{th}}$ binary KRI are $p_{j1}$=50% and $p_{j2}$=30%, respectively. These estimates are derived from historical data of similar products, medical and statistical considerations, or other available criteria.

It should be noted that for certain distributions, risk level estimates may need to be adjusted based on the time of risk-based monitoring. For instance, if historical data indicates a high-risk incidence proportion of 50% for a binary KRI after 2 years of follow-up, using this criterion as a high-risk level estimate for risk-based monitoring conducted after 2 months of follow-up may not be appropriate. Hence, two approaches can be used to determine the estimates of risk levels at different risk-based monitoring times. The first approach involves using specified rules. For example, the high-risk discontinuation rate of each risk-based monitoring is defined as 30% more than the average discontinuation rate across all sites. The second approach does not rely on the average value but rather adjusts the risk level estimates based on different monitoring times. This typically requires certain assumptions and is only applicable for the risk boundaries based on the Poisson process and binomial distribution; the details of the technique are presented in Calculating the optimal risk boundaries and Binomial, exponential and normal distributions sections, respectively.

### Calculating the optimal risk boundaries

We use the Poisson-distributed site-level KRIs to illustrate how to calculate the optimal risk boundaries, as the derivation for other distributions or levels is similar. Thus, according to Notations for the observed KRIs’ outcomes section, we first need to define the observed KRI’s outcomes $X_{jk} \sim \text {Poisson}(\Lambda _{jk})$. As mentioned in Establishing risk levels for KRIs along with their corresponding monitoring actions and estimates section, we next need to define the $g^{\text{th}}$ risk-level estimate following a Poisson distribution $\text {Poisson}(\lambda _{jg})$, where $\lambda _{jg}$ denote the estimated average number of events of the $j^{\text{th}}$ KRI per subject in a unit time at the $g^{\text{th}}$ risk level. $g={1,2,\dots ,G}$ and $\lambda _{j1}>\lambda _{j2}>\dots >\lambda _{jG}$.

In this step, our objective is to find the optimal risk boundary, denoted as $\theta _{jgk}$, between the $g^{\text{th}}$ and $(g+1)^{\text{th}}$ risk level estimates based on the $j^{\text{th}}$ Poisson-distributed KRI at the $k^{\text{th}}$ site. To achieve this, one needs to establish a total of *G* hypotheses whenever we conduct risk-based monitoring for the $j^{\text{th}}$ KRI at the $k^{\text{th}}$ site; for example, $\text{H}_{jgk}: \Lambda _{jk}=\lambda _{jg}$, indicating that the true risk of the $k^{\text{th}}$ site based on the $j^{\text{th}}$ KRI is at the $g^{\text{th}}$ risk level. Our method determines the optimal risk boundary $\theta _{jgk}$ by minimising the decision error rate, which is given by

1 $$\begin{aligned} \text {Pr}(\text {Error}){} & {} = \sum \limits _{g=1}^{G} \text {Pr}(\text {H}_{jgk})\text {Pr}( \overline{S}_g|\text {H}_{jgk}) \nonumber \\{} & {} = \sum \limits _{g=1}^{G-1} \{\text {Pr}(\text {H}_{jgk})\text {Pois}(\theta _{jgk};\lambda _{jg})- \text {Pr}(\text {H}_{jgk})\text {Pois}(\theta _{jgk};\lambda _{j(g+1)})\}+ \sum \limits _{g=2}^{G}\text {Pr}(\text {H}_{jgk}), \end{aligned}$$

where $\overline{S}_g$ represents the complement of $S_g$ and $S_g$ denotes the monitoring action to be taken at the $g^{\text{th}}$ risk level. To avoid clutters, we abuse the notation for probabilities without specifying the observed value of the underlying random variable. As can be seen from Eq. (1), our general framework can treat different hypotheses as random events. More concretely, $\text {Pr}(\overline{S}_g|\text{H}_{jgk})$ indicates the probability when the true risk of the $k^{\text{th}}$ site is at the $g^{\text{th}}$ level, yet we do not take the correct monitoring action. $\text {Pr}(\text{H}_{jgk})$ represents the prior probability that the $g^{\text{th}}$ hypothesis is true. Hence, in Eq. (1), the decision error rate that will be minimised is the posterior probability of incorrect decisions, a simple calculation using the Bayes’ rule. $\text {Pois}(\theta _{jgk};\lambda _{jg})=\sum \nolimits _{\tau _{jk}=0}^{\lfloor \theta _{jgk}\rfloor }e^{-\lambda _{jg}}\frac{(\lambda _{jg})^{\tau _{jk}}}{\tau _{jk}!}$ is the cumulative mass function of the Poisson distribution when the number of events is below $\lfloor \theta _{jgk} \rfloor$, where $\tau _{jk}$ denotes the average number of events of the $j^{\text{th}}$ KRI per subject at the $k^{\text{th}}$ site. Appendix A demonstrates that due to the monotonicity of the optimisation objective function, the minimum decision error rate is achieved when $\tau _{jk}$ is at its maximum. By minimising the decision error rate (in close-form), we get the analytical solution for the optimal risk boundary $\theta _{jgk}$:

2 $$\begin{aligned} \text {Poisson distribution:} \quad \theta _{jgk}=\frac{\ln \left( \frac{\Pr (\text {H}_{j(g+1)k})}{\Pr (\text {H}_{jgk})}\right) }{\ln \lambda _{jg}-\ln \lambda _{j(g+1)}}+\frac{\lambda _{jg}-\lambda _{j(g+1)}}{\ln \lambda _{jg}-\ln \lambda _{j(g+1)}}. \end{aligned}$$

The two terms in the above display represent the influence of prior information and KRIs’ risk level estimates on the determination for the risk boundaries. It can be interpreted as a non-informative prior when setting $\Pr (\text {H}_{jgk})=\Pr (\text {H}_{j(g+1)k})$ almost surely. A total of $G - 1$ risk boundaries can be identified based on *G* different risk levels. Compared to all other values between the two risk levels, the risk boundary we found minimises the decision error rate, so we named it the optimal risk boundary.

In addition, there are two other points that can be expanded upon. First, consider a scenario where there are a total of *J* independent Poisson-distributed KRIs at the site-level. Since the family of Poisson distributions is closed under summation, we have $X_k\sim \text {Poisson}(\Lambda _k)$, where $X_k=\sum \nolimits _{j=1}^J X_{jk}$ and $\Lambda _k= \sum \nolimits _{j=1}^J \Lambda _{jk}$. Similarly, we can calculate the sum of $\lambda _{jg}$ as $\lambda _g=\sum \nolimits _{j=1}^J \lambda _{jg}$ and use this value as the risk level estimate in place of $\lambda _{jg}$ to calculate the risk boundary $\theta _{gk}$, where $\lambda _1>\lambda _2>\cdots >\lambda _G$. Consequently, we can evaluate the risk level of the $k^{\text{th}}$ site using single or multiple KRIs. Second, in the Poisson distribution, we convert the collected data to a unit time scale to compare the average number of events. Another approach is to keep the data unchanged and adjust the estimates of risk levels based on the risk-based monitoring times. This allows for the comparison of the average total number of events per subject until time *t*, which is denoted by $N_{jk}(t)$. In this context, $\{N_{jk}(t),t\ge 0\}$ is a counting process. When it satisfies the conditions for a Poisson point process $P\{{N_{jk}(t)-N_{jk}(0)=\tau _{jk}}\}$ [12], and considering *J* KRIs in total, we obtain the following expression for the decision error rate when the KRIs follow a Poisson process:

3 $$\begin{aligned} \text {Pr}(\text {Error}){} & {} = \sum \limits _{g=1}^{G} \text {Pr}(\text {H}_{gk})\text {Pr}( \overline{S}_g|\text {H}_{gk}) \nonumber \\{} & {} = \sum \limits _{g=1}^{G-1} \{\text {Pr}(\text {H}_{gk})\text {P}(\Theta _{gk};\lambda _{g}t_{k})- \text {Pr}(\text {H}_{gk})\text {P}(\Theta _{gk};\lambda _{g+1}t_{k})\}+ \sum \limits _{g=2}^{G}\text {Pr}(\text {H}_{gk}), \end{aligned}$$

where $\text{H}_{gk}: \Lambda _{k}t_{k}=\lambda _{g}t_{k}$, and by abuse of notation $\Lambda _k t_k := \sum \nolimits _{j=1}^J\Lambda _{jk}t_{jk}$ and $\lambda _g t_k := \sum \nolimits _{j=1}^J\lambda _{jg}t_{jk}$. Here $t_{jk}$ denotes the average follow-up time from enrollment or the previous risk-based monitoring to the current risk-based monitoring for the $j^{\text{th}}$ KRI at the $k^{\text{th}}$ site. Typically, $t_{1k},t_{2k},\ldots ,t_{Jk}$ are the same because each KRI often shares the same follow-up time at the same site. Furthermore, $\text {P}(\Theta _{gk};\lambda _g t_k)=\sum \nolimits _{\tau _k=0}^{\lfloor \Theta _{gk}\rfloor }\text {P}\{N_k(t_k)-N_k(0)=\tau _k\}=\sum \nolimits _{\tau _k=0}^{\lfloor \Theta _{gk}\rfloor }e^{-\lambda _g t_k}\frac{(\lambda _g t_k)^{\tau _k}}{\tau _k!}$, where $N_k(t) :=\sum \nolimits _{j=1}^JN_{jk}(t)$ and $\tau _k :=\sum \nolimits _{j=1}^J\tau _{jk}$. $\Theta _{gk}$ represents the optimal risk boundary based on the Poisson process at the $g^{\text{th}}$ level and the $k^{\text{th}}$ site. Using the same method as that for getting $\theta _{jgk}$, we obtain:

4 $$\begin{aligned} \text {Poisson process:} \quad \Theta _{gk}=\frac{\ln \left( \frac{\Pr (\text {H}_{(g+1)k})}{\Pr (\text {H}_{gk})}\right) }{\ln \lambda _g t_k-\ln \lambda _{g+1}t_k}+\frac{\lambda _g t_k-\lambda _{g+1}t_k}{\ln \lambda _g t_k-\ln \lambda _{g+1}t_k}. \end{aligned}$$

The derivation of Eqs. (2) and (4) can be found in Appendix A.

### Comparing and making decisions

Consider a situation where we categorise KRIs into three risk levels, whose monitoring actions correspond to that on-site monitoring is required, recommended, or unnecessary, respectively. Through Eq. (4), we can obtain two risk boundaries, denoted as $\Theta _{1k}$ and $\Theta _{2k}$. Let $\hat{E}_{jk}$ be the average observed number of events of the $j^{\text{th}}$ KRI per subject at the $k^{\text{th}}$ site when conducting risk-based monitoring. We calculate $\hat{E}_{k} := \sum \nolimits _{j=1}^J\hat{E}_{jk}$ and compare it against $\Theta _{1k}$ and $\Theta _{2k}$ to decide if on-site monitoring is warranted. If $\hat{E}_k\le \Theta _{2k}$, it indicates that on-site monitoring can be temporarily stopped for the $k^{\text{th}}$ site since the KRIs are at a low-risk level. If $\Theta _{2k}<\hat{E}_k\le \Theta _{1k}$, on-site monitoring is recommended if there is sufficient budget or resources. Otherwise, if $\hat{E}_k>\Theta _{1k}$, on-site monitoring is mandated. $\Theta _{gk}$ is only determined by $t_{jk}$ and $\lambda _{jg}$; it is not affected by the sample size. Notably, for multiple KRIs, in addition to comparing the combined KRI against the risk boundary $\Theta _{gk}$, another feasible way is to assess the risk level of the $j^{\text{th}}$ KRI based on $\Theta _{jgk}$ and establish a criterion for monitoring actions, where $\Theta _{jgk}$ is the risk boundary calculated based on the $j^{\text{th}}$ KRI only. For example, if five or more of the ten KRIs at the $k^{\text{th}}$ site are assessed as high-risk, on-site monitoring should be conducted.

When conducting risk assessments using multiple KRIs, the importance of each KRI may be different, or we may have less confidence in risk level estimates for certain KRIs. Therefore, one strategy is to incorporate weighting coefficients when summing over different KRIs. That is, we could redefine $\lambda _{g} t_{k}$ as $\lambda _{g}t_{k} := \sum \nolimits _{j=1}^J w_j \lambda _{jg} t_{jk}$. This flexibility allows us to assign more weights to important KRIs or KRIs with better estimates. When $w_1=\cdots =w_J=1$, $\lambda _{g}t_{k}$ reduces to the unweighted version. On the other hand, the $j^{\text{th}}$ KRI has no influence on $\lambda _{g}t_{k}$ when $w_j=0$. Commonly used weighting options can be based on: a fully data-driven approach by calculating the Euclidean distance between two neighbouring risk level estimates of each KRI [5, 11], or simply prior experience, knowledge, or even some subjective choices on a case-by-case basis. When using the weighting method, $\hat{E}_{k} :=\sum \nolimits _{j=1}^Jw_j\hat{E}_{jk}$ and each KRI in $\hat{E}_k$ should be assigned the same weights as those in $\lambda _{g}t_{k}$. This ensures that the comparison between $\hat{E}_k$ and $\Theta _{gk}$ remains meaningful.

### Binomial, exponential and normal distributions

We do not limit ourselves just to the Poisson distribution, because other distributions such as the binomial, exponential, or normal distribution are also very common in applications. Normal distribution is still additive and the previous weighting strategy for Poisson distribution can be straightforwardly extended to normal distribution. The additivity of the binomial distribution requires two extra conditions: first, the true incidence proportions of multiple KRIs at the same site are the same, and second, the same risk level estimates of these KRIs are also the same. However, when summing over multiple independent exponential-distributed KRIs, they no longer follow the exponential distribution but instead follow the Gamma distribution. But this issue can be rectified by considering the Gamma distribution instead, which we do not further pursue in this paper. Assuming there are *J* KRIs in total, the optimal risk boundaries for these three distributions are given by

5 $$\begin{aligned}{} & {} \text {Binomial distribution:} \quad \pi _{gk} = \frac{n_k^{-1}\ln \left( \frac{\text {Pr}(\text {H}_{(g+1)k})}{\text {Pr}(\text {H}_{gk})}\right) }{\ln \left( \frac{p_g(1-p_{g+1})}{p_{g+1}(1-p_g)}\right) } + \frac{\ln \left( \frac{1-p_{g+1}}{1-p_g}\right) }{\ln \left( \frac{p_g(1-p_{g+1})}{p_{g+1}(1-p_g)}\right) }, \end{aligned}$$

6 $$\begin{aligned}{} & {} \text {Exponential distribution:} \quad e_{jgk} = \frac{\ln \left( \frac{\text {Pr}(\text {H}_{j(g+1)k})}{\text {Pr}(\text {H}_{jgk})}\right) }{\lambda _{jg}-\lambda _{j(g+1)}} + \frac{\ln \lambda _{jg}-\ln \lambda _{j(g+1)}}{\lambda _{jg}-\lambda _{j(g+1)}}, \end{aligned}$$

7 $$\begin{aligned}{} & {} \text {Normal distribution:} \quad \delta _{gk} = \frac{\ln \left( \frac{\text {Pr}(\text {H}_{(g+1)k})}{\text {Pr}(\text {H}_{gk})}\right) \sigma ^2}{\mu _g-\mu _{g+1}} + \frac{\mu _g+\mu _{g+1}}{2}, \end{aligned}$$

where we assume that estimates of KRIs for these three distributions at the $g^{\text{th}}$ risk level follow $\text {Bin}(p_{g},n_k)$, $\text {Exp}(\lambda _{jg})$, and $\mathcal {N}(\mu _g,\sigma _g^2)$, respectively. $n_k=\sum \nolimits _{j=1}^J n_{jk}$ and $p_{1g}=p_{2g}=\dots =p_{Jg}=p_{g}$. $\mu _g=\sum \nolimits _{j=1}^J w_j \mu _{jg}$ and $\sigma _1^2=\sigma _2^2=\dots =\sigma _G^2=\sum \nolimits _{j=1}^J \sigma _{jg}^2=\sigma ^2$. $\text{H}_{gk}$ in Eqs. (5) and (7) represent $P_{k}=p_{g}$ and $M _k=\mu _g$, respectively. $\text{H}_{jgk}$ in Eq. (6) represents $\Lambda _{jk}=\lambda _{jg}$.

For the binomial distribution, since the risk boundaries use multiple binary KRIs, the scenario that sites with a small number of subjects may have a high incidence proportion undoubtedly decreases unless multiple low-risk KRIs occur at the same time. However, $\pi _{gk}$ does not consider the influence of follow-up time. In addition to using some specified rules mentioned in Establishing risk levels for KRIs along with their corresponding monitoring actions and estimates section, the risk boundaries of the binomial distribution can also be adjusted based on the risk-based monitoring time. Consider the following scenario: let $t_{jg}$ denote the average follow-up time for the $j^{\text{th}}$ KRI in historical data, such as 2 years in Establishing risk levels for KRIs along with their corresponding monitoring actions and estimates section. When the occurrence time of events for the $j^{\text{th}}$ KRI follows an exponential distribution, we obtain $\lambda _{jg} = -\frac{\ln (1-p_{jg})}{t_{jg}}$, where $\lambda _{jg}$ and $p_{jg}$ are defined identically as in Establishing risk levels for KRIs along with their corresponding monitoring actions and estimates section. Next, when conducting risk-based monitoring, the average follow-up time from enrollment or the previous risk-based monitoring to the current risk-based monitoring for the $j^{\text{th}}$ KRI at the $k^{\text{th}}$ site is denoted as $t_{jk}$, such as 2 months in Establishing risk levels for KRIs along with their corresponding monitoring actions and estimates section. We use the equation $p_{jgk}=1-\exp (-\lambda _{jg} t_{jk})$ to calculate an updated parameter $p_{jgk}$, which is the adjusted incidence proportion of the $j^{\text{th}}$ KRI at the $g^{\text{th}}$ risk level and the $k^{\text{th}}$ site based on follow-up time $t_{jk}$. Finally, we use $p_{jgk}$ in place of $p_g$ in Eq. (5) to calculate the risk boundary $\pi _{jgk}$. Furthermore, under the non-informative prior condition, when the $j^{\text{th}}$ KRI can only occur once, if we calculate $\Theta _{jgk}$ based on $\lambda _{jg}$ and $t_{jk}$, then convert $\Theta _{jgk}$ to $\pi _{jgk}^{*}$ using the formula $\pi _{jgk}^{*}=1-\exp (-\Theta _{jgk})$. We observe that $\pi _{jgk}^{*}$ and $\pi _{jgk}$, which are the $j^{\text{th}}$ KRI’s risk boundaries derived from the Poisson process and the binomial distribution, respectively, are very close. The former increasingly converges to the latter as the difference between two neighbouring risk level estimates decreases. Through simulations, it was found that the difference between the two is less than 0.01 in most cases. See Fig. S1 in Appendix C for more details. In addition to adjusting the risk boundary of the binomial distribution based on follow-up time, the aforementioned method can also address the limitation of Eq. (5) for handling multiple KRIs at the same risk level but with different incidence proportions. We can convert their incidence proportions or the average of them to incidence rates, then calculate the risk boundary based on the Poisson process and conduct risk assessments.

For the same $\lambda _{jg}$, the exponential distribution’s risk boundary can be derived as the reciprocal of the Poisson distribution’s risk boundary when there is only one KRI and a non-informative prior is used. For the normal distribution, Eq. (7) shows that its optimal risk boundary is the median of two neighbouring KRIs’ risk level estimates ($\frac{\mu _g+\mu _{g+1}}{2}$) when using a non-informative prior. Similar findings can be observed when assuming that a binary KRI follows a uniform distribution. Only when the sum of two neighbouring risk level estimates equals 1, the risk boundary based on a binomial distribution equals the median. In terms of comparison, for the binomial or normal distribution, we can directly compare the observed results against their risk boundaries, while we need to convert the risk boundaries by multiplying them with $-\ln ({Pr_{jk}})$ for the exponential distribution, where $Pr_{jk}$ represents the percentage of subjects at the $k^{\text{th}}$ site who have experienced the $j^{\text{th}}$ KRI event when conducting risk-based monitoring. Subsequently, we compare them against the observed quantile survival time, such as the first quartile (*Q*1) survival time when $Pr_{jk}=0.25$. A summary of the optimal risk boundaries by distribution is provided in Appendix F.

### Minimise the average decision error rate

Consider a hypothesis that the risk at the $k^{\text{th}}$ site falls within a specific risk interval, such as $\text{H}_{gk}{:}\lambda _{g}\le \Lambda _k<\lambda _{g-1}$. In this situation, the optimal risk boundaries are determined by minimising the average decision error rate. As an illustration, for the Poisson distribution,

8 $$\begin{aligned} \text {Pr}(\text {Error}){} & {} = \sum \limits _{g=2}^{G} \text {Pr}(\text {H}_{gk}) \int _{\lambda _{g}}^{\lambda _{g-1}} f(\Lambda _k|\text {H}_{gk}) \text {Pr}( \overline{S}_{g-1}|\text {H}_{gk})d\Lambda _k \nonumber \\{} & {} = \sum \limits _{g=2}^{G-1} \sum \limits _{\tau =0}^{\lfloor \theta _{(g-1)k} -1\rfloor } \left[ f(\tau ) \{ \text {Pr}(\text {H}_{gk}|\tau ) - \text {Pr}(\text {H}_{(g+1)k}|\tau ) \}\right] + \sum \limits _{g=2}^{G}\text {Pr}(\text {H}_{gk}), \end{aligned}$$

where $G\ge 3$. $f(\Lambda _k|\text {H}_{gk})$ denotes the probability of $\Lambda _k \in (\lambda _{g},\lambda _{g-1})$. Typically, it is assumed to follow the uniform distribution $U(\lambda _{g},\lambda _{g-1})$. $\tau$ denotes the average number of events per subject. $f(\tau )$ is the probability density function of $\tau$. The remaining parameters’ definitions are similar to those in Calculating the optimal risk boundaries section. We have

9 $$\begin{aligned} \text {Pr}(\text {H}_{gk} \mid \tau ) = \dfrac{\text {Pr}(\text {H}_{gk}) \{\text {Pois}(\tau +1;\lambda _{g-1})-\text {Pois}(\tau +1;\lambda _g)\}}{f(\tau )(\lambda _{g-1}-\lambda _g)}, \end{aligned}$$

where $\text {Pois}(\tau +1;\lambda _g)$ represents the cumulative mass function of the Poisson distribution. The complete derivation for the Poission and other distributions can be found in Appendix B. When minimising the average decision error rate, the optimal risk boundaries have no analytical solution and need to be solved numerically.

### Non-constant risk rate

In the previous sections, we assumed that the incidence rate used in the Poisson or exponential distribution and the incidence proportion used in the binomial distribution do not vary over time. However, according to Liu et al. [13], this assumption is typically applicable only to rare events. Furthermore, if we take action on high-risk KRIs, it is typical for the risk rates of KRIs to differ before and after the intervention. Therefore, we adopt the approach proposed by Zink et al. [21], which divided the time into fixed-duration windows and assessed the risk separately within each window. The division of time windows can be based on two patterns: calendar days and study days. The former is based on the actual length between two dates, while the latter is based on the time relative to the date of the first dose. For example, a high discontinuation rate may occur in a certain time window divided based on calendar days due to COVID-19 pandemic-related reasons. On the other hand, if the incidence rate of an adverse event (AE) changes with the cumulative administration of the treatment drug, then time windows based on study days may be appropriate. For example, in trials of immune checkpoint inhibitors, when a sufficient number of immune cells are activated to have effects, some immune-related AEs may occur at the same time. In this situation, we can divide time windows based on the points where efficacy changes [19].

The risk estimate of each time window’s can also be determined through the two approaches mentioned in Establishing risk levels for KRIs along with their corresponding monitoring actions and estimates section. Additionally, using an informative prior can contribute to adjusting risk boundaries. For instance, in a time window with a high discontinuation rate due to the COVID-19 pandemic, setting $\text{Pr}(\text{H}_{(g+1)k})>\text{Pr}(\text{H}_{gk})$ can raise the risk boundary. This indicates that although the discontinuation rate is relatively high in certain time windows, sponsors believe that this is a consequence of the COVID-19 pandemic rather than trial quality issues. Thus, a higher risk boundary is set compared to that in other time windows. In Example section, we provide a more detailed explanation on how prior information affects the calculation of risk boundaries.

## Simulation

In Simulation and Results section, we first verified whether the proposed method is consistent with theoretical reasoning that can find the optimal risk boundaries within a given range of risk levels. Here we conducted simulations with a single binary KRI $(J=1)$. We designed each simulation to involve only one site, with all subjects being recruited at the same time. We established high- or low-risk levels. Each site was randomly assigned to high- or low-risk levels in a 1:1 ratio and enrolled 100 subjects. The reason for using such a large sample size is to obtain stable results to demonstrate the optimality of the proposed risk boundaries. Additionally, we simulated a scenario in which there were 50 subjects and the ratio of low- to high-risk sites was 7:3. We used this allocation ratio as prior information when calculating the risk boundaries. We defined six high- ($g=1$) and low-risk ($g=2$) estimate ($p_{jg}$) scenarios, whose ($p_{11}$, $p_{12}$) were (15%, 5%), (20%, 10%), (20%, 15%), (25%, 15%), (35%, 25%), and (40%, 30%), respectively. The occurrence times of all KRI events were sampled from an exponential distribution $\text {Exp}(\lambda _{jg})$ whose parameter $\lambda _{jg}$ was converted from the incidence proportion $p_{jg}$ using the equation $\lambda _{jg} = -\frac{\ln (1-p_{jg})}{t_{jg}}$, where $t_{jg}=10$. By comparing the occurrence time of the event with the risk-based monitoring time, we determined if the KRI event occurred. The risk-based monitoring time in this simulation was set for the $\text{10}^{\text{th}}$ month after the first dose.

Furthermore, we compared the results of the proposed risk boundary with those of directly using the median of two neighbouring risk level estimates. In this simulation, the KRI events in the high- and low-risk sites were sampled from a binomial distribution $\text {Bin}(p_{jg},n)$ with incidence proportions of 30% ($p_{11}$) and 10% ($p_{12}$), and the total number of subjects (*n*) were 10, 20, and 50, respectively.

Next, we simulated 12 scenarios to further identify the influencing factors for the proposed risk boundaries. Table 1 summarises the parameters used in these simulations, including the number of sites, total number of subjects (*n*), percentage of collected monitoring data, and either the incidence proportion ($p_{jg}$) or the average number of KRI events ($\lambda _{jg}$). Except in Scenarios 9 and 10, the events of KRIs in all other scenarios can only occur once. The simulation data for Scenarios 9 and 10 was sampled from $\text {Poisson}(\lambda _{jg})$. For all other scenarios, we used the same pattern as in the previous simulations to sample the occurrence time from the exponential distribution and compare it with the risk-based monitoring time to determine whether the event occurred. When there was a single site, all subjects were recruited at the same time, with $t_{jg}=10$. When there were 5 and 10 sites, we simulated the multicenter competitive recruiting scenarios. We set an accrual period (*a*) and assigned the recruitment time ($a \cdot U$) to each subject, where $U \sim \text{Unif} (0,1)$. For 5 and 10 sites, the number of subjects for each site was sampled from the multinomial distributions $\text {PN} (n,0.25,0.25,0.2,0.2,0.1)$ and $\text {PN} (n,0.15,0.15,0.15,0.1,0.1,0.1,0.1,0.05,0.05,0.05)$, respectively. $t_{jg}$ in multi-center trials equals $f-\frac{a}{2}$, where *f* represents follow-up period.

**Table 1:** Summary of 12 simulation scenarios with various parameters

Scenario	# of site	Total # of subjects ($\varvec{n}$)	% of Monitoring^a^	Incidence proportion ($\varvec{p}_{\varvec{jg}}$) or average number of KRI events ($\varvec{\lambda }_{\varvec{jg}}$) at different risk levels
1	1	1-25	10%-100%	$p_{11}=40\%$, $p_{21}=25\%$, $p_{31}=10\%$,
				$p_{12}=10\%$, $p_{22}=4\%$, $p_{32}=1\%$
2	1	1-25	10%-100%	$p_{11}=40\%$, $p_{21}=30\%$, $p_{31}=20\%$,
				$p_{12}=25\%$, $p_{22}=15\%$, $p_{32}=5\%$
				$p_{13}=10\%$, $p_{23}=4\%$, $p_{33}=1\%$
3	1	1-25	10%-100%	$p_{11}=20\%$, $p_{21}=20\%$, $p_{31}=5\%$,
				$p_{12}=20\%$, $p_{22}=5\%$, $p_{32}=1\%$
4^b^	1	1-25	10%-100%	Same as Scenario 3
5	5	20,50,80	33%	Same as Scenario 1
6	5	120	20%	Same as Scenario 1
7	10	160,200	33%	Same as Scenario 1
8	10	200,400	20%	Same as Scenario 1
9	5	20,30	33%	$\lambda _{11}=7$, $\lambda _{21}=4$, $\lambda _{31}=1$,
				$\lambda _{12}=5$, $\lambda _{22}=3$, $\lambda _{32}=1$
10	10	50,80	20%	Same as Scenario 11
11	1	1-25	30%,50%	$p_{11}=25\%$, $p_{12}=10\%$
12	1	1-25	30%,50%	$p_{11}=35\%$, $p_{21}=25\%$, $p_{31}=15\%$,
				$p_{12}=15\%$, $p_{22}=10\%$, $p_{32}=5\%$

^a^represents risk-based monitoring is conducted when a certain percentage of the trial data is collected

^b^indicates that the scenarios were weighted, and the weights ($w_{p_{jg}}$) assigned to different incidence proportions are $w_{p_{1g}}=10\%$, $w_{p_{2g}}=45\%$, and $w_{p_{3g}}=45\%$ respectively

The sites in Scenario 2 were categorised into high-, medium-, and low-risk levels, while all other scenarios only had high- and low-risk levels. The allocation ratio between high-risk and low-risk sites was 2:3 in Scenarios 5, 6, and 9, 3:2 in Scenarios 7, 8, and 10, and 1:1 in the remaining scenarios. We simulated the scenario where some incidence proportions were the same at different risk levels, such as in Scenario 3. Also, in Scenario 4, we weighted incidence proportions to evaluate if such weighting would improve performances compared to the unweighted Scenario 3 with identical parameters. Each scenario had 3 KRIs, except for Scenarios 11 and 12. To be more similar to the real situation, even at the same risk level, the incidence proportions of different KRIs varied. For instance, in Scenario 1, the incidence proportions of different KRIs at the high-risk site ($p_{j1}$) were 40%, 25%, and 10%, respectively. Lastly, we evaluated the influence of various numbers of KRIs on performance in Scenarios 11 and 12. Scenario 11 used only one KRI, while Scenario 12 used 3, 5, and 10 KRIs, respectively. The ratios of different incidence proportions at the same risk level were 1:1:1 for 3 KRIs and 2:1:2 for 5 and 10 KRIs. These allocation ratios ensured that in Scenarios 11 and 12, regardless of the number of KRIs involved, the average incidence proportions at the same risk level were equal. The evaluation metric was the accuracy rate for the site risk assessment. In the simulations, the non-informative prior risk boundaries were calculated based on the Poisson process, while the informative prior risk boundaries were calculated based on the binomial distribution. In each simulation scenario, 1000 replications were generated. All the simulation studies are done in software SAS 9.4.

## Results

**Fig. 1:**
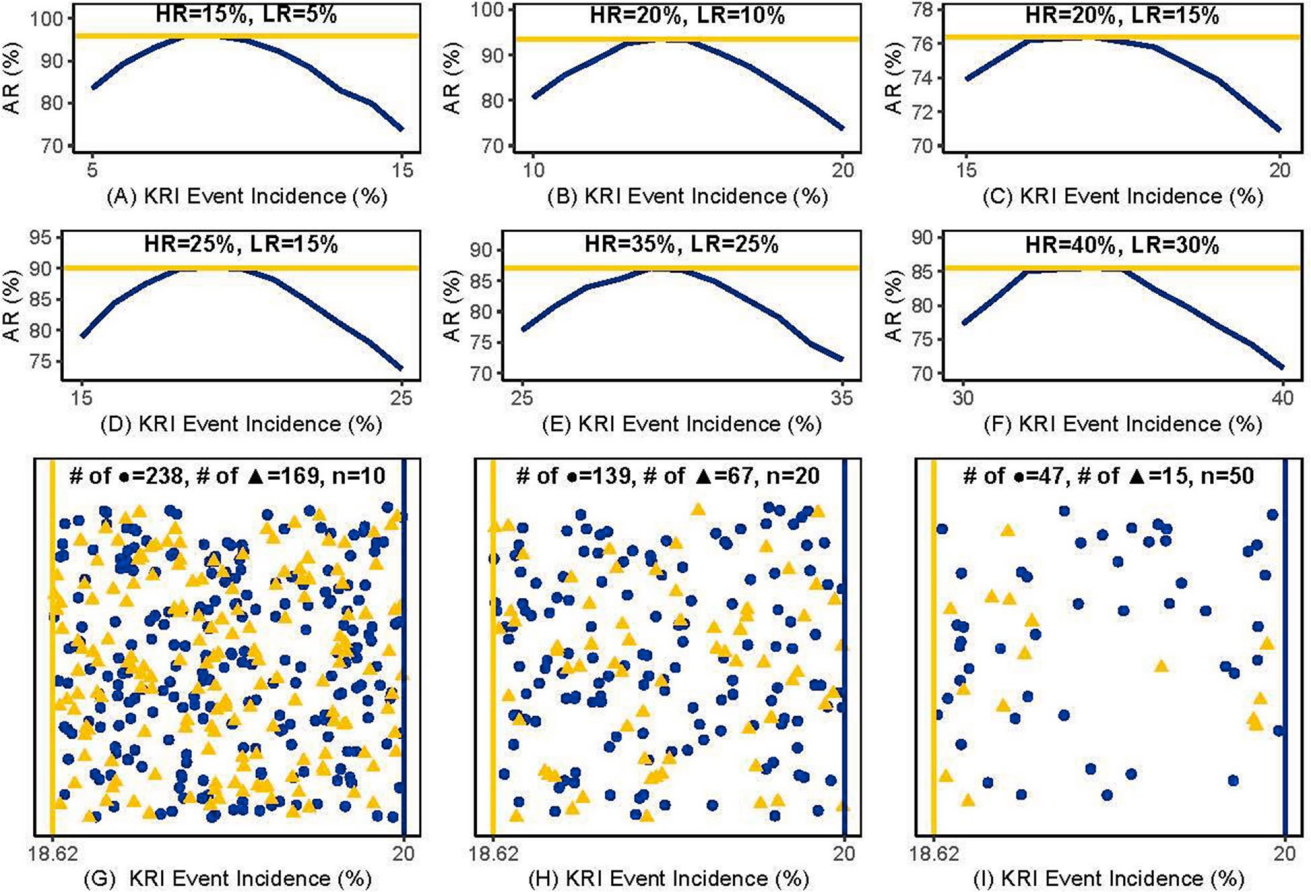
The comparison of the performances between the proposed risk boundaries and all other values within the incidence proportion range, including the median. HR and LR denote high- and low-risk levels, respectively. AR is the abbreviation for accuracy rate

The accuracy rates of the risk boundaries obtained using the proposed method are shown by the gold lines in Panels A-F of Fig. 1, while the blue lines represent the accuracy rate using all other values within the range of high- and low-risk levels. The simulation results demonstrated that the proposed risk boundaries found the highest accuracy rate, while the median did not achieve the highest accuracy rate for the binary KRI in most scenarios. In the non-informative prior, the proposed risk boundary is typically lower than the median if the latter is below 50%. In contrast, when the median exceeds 50%, the proposed risk boundary is higher. They overlap when the median is 50%. The exception occurred when informative priors were used. The results where the ratio of high-risk to low-risk sites was not 1:1 are provided in Appendix D. The accuracy rates with and without prior information are represented by the gold lines and the black lines in the figure, respectively. Similarly to the non-informative prior, the proposed method identified the optimal risk boundaries.

When considering incidence proportions of 30% and 10% for high- and low-risk levels, respectively, the Panels G-I in Fig. 1 show the differences between the proposed risk boundary (18.62%) and the median (20%) with various numbers of subjects. To make the results clear and easy to understand, we only drew sites that fell within this interval and randomly moved the data points in the event of unaltered accuracy. Outside of this interval, the performances of the two were the same. When using the median, sites with a true incidence proportion of 30% were misclassified as low-risk, as represented by the dots in the figure, whereas our method correctly identified them as high-risk. Conversely, when sites with a true incidence proportion of 10% fell within this interval, it indicated that the median correctly evaluated the risk while our method was wrong, as represented by the triangles. The final results revealed that the proposed risk boundaries exhibited 60-70 fewer mistakes in 1000 simulations than using the median when the total number of subjects was 10 or 20. When there were 50 subjects, fewer sites fell within this interval due to the increase in the sample size, yet the proposed risk boundaries still demonstrated an approximately 3% higher accuracy rate than the median.

**Fig. 2:**
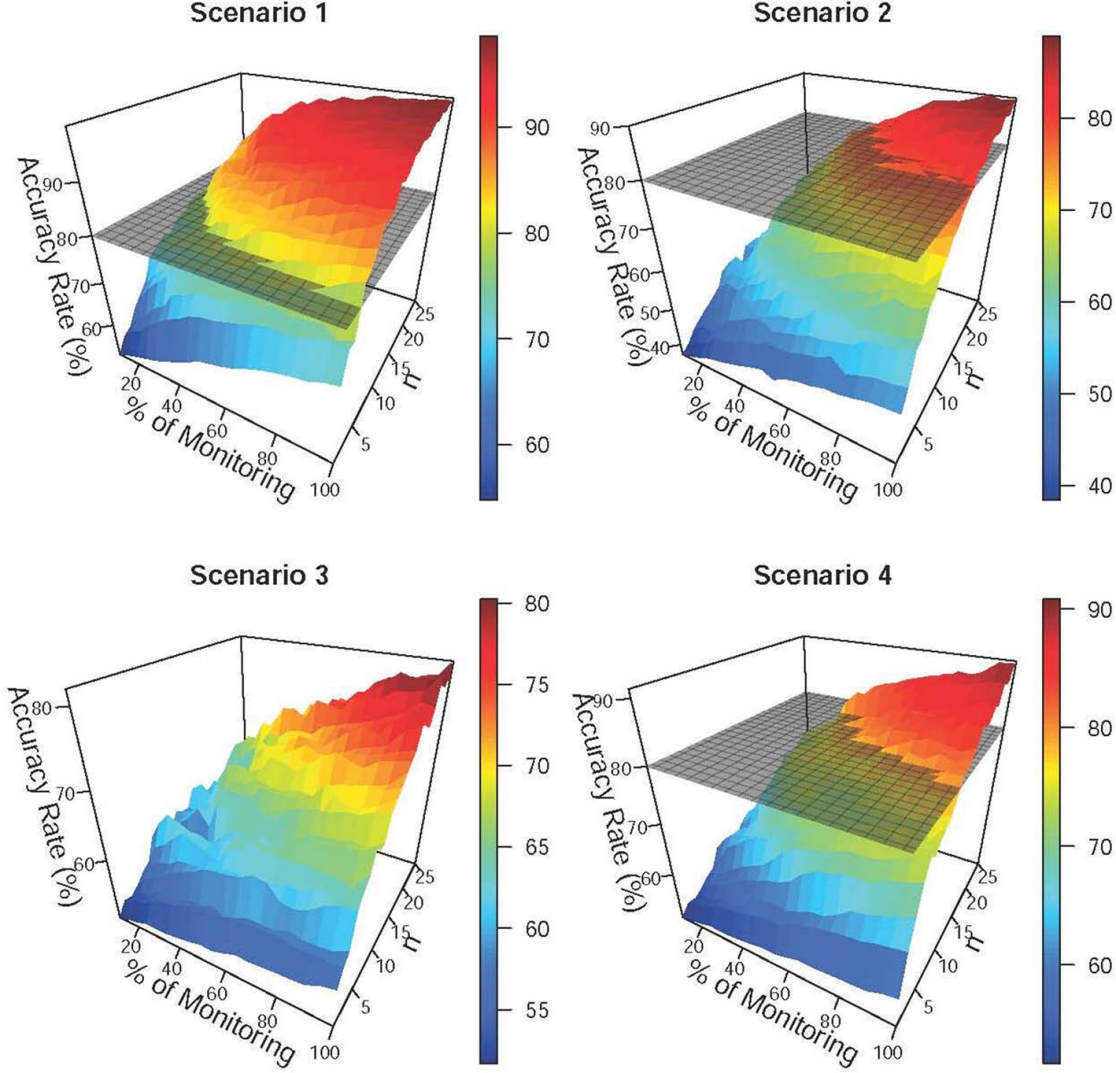
The influence of various percentages of monitoring and the number of subjects on the accuracy rate

Figure 2 shows how the accuracy rate varies with the percentage of monitoring and the total number of subjects. The grey, transparent plane represents an accuracy rate of 80%. The accuracy rates in Scenario 1 were higher than 80% in most parts. Impacted by the diminishing gap between different risk level estimates, the accuracy rates in Scenario 2 were higher than 80% only when there were a substantial number of subjects or monitoring data. Scenario 4 used a weighting strategy and improved accuracy rates compared to Scenario 3, thus demonstrating the viability of the weighting strategy.

In the case of 5 sites, such as Scenarios 4 and 5 in Table 2, the accuracy rate was high when there were more subjects and at least 33% of the monitoring data was collected. Otherwise, the accuracy rate would decrease if either of these conditions were not satisfied. Similar findings were observed when there were 10 sites. For example, the accuracy rate was 82.6% in Scenario 7, where the total number of subjects was 200 and 33% of the monitoring data was collected. On the other hand, the accuracy rate was 81.3% when only 20% of the monitoring data was collected in Scenario 8, even though there were 400 subjects. The above findings remind us that, similar to single site simulation results, the accuracy rate of multicenter simulations was also influenced by the quantity of monitoring data and the total number of subjects. The duration of the accrual period and follow-up period also affected the accuracy rate. This is because a lower amount of censored data is associated with a shorter accrual period or a longer follow-up period. In scenarios where the number of sites differed while keeping other factors constant, such as Scenario 5 with 80 subjects and Scenario 7 with 160 subjects, the subjects were more dispersed due to the increase in site number. Consequently, the accuracy rate was impacted to some extent. At last, when KRIs could occur multiple times, the simulation results also complied with the above-mentioned rules.

**Table 2:** The accuracy rate of the proposed risk boundaries in multicenter scenarios

Scenario (% of M^a^)	# of site	$\varvec{n}$	Accrual Period ($\varvec{a}$)	Follow-up Period ($\varvec{f}$)	Accuracy Rate
5 (33%)	5	20	1	3	73.8%
		50	3	7	79.8%
		80	4	8	84.3%
6 (20%)	5	120	10	10	79.9%
7 (33%)	10	160	4	8	79.7%
		200	6	12	82.6%
8 (20%)	10	200	6	12	72.7%
		400	8	16	81.3%
9 (33%)	5	20	NA	NA	80.3%
		30	NA	NA	84.9%
10 (20%)	10	50	NA	NA	81.1%
		80	NA	NA	86.3%

^a^ represents the percentages of monitoring. The units of accrual period and follow-up period are months

**Fig. 3:**
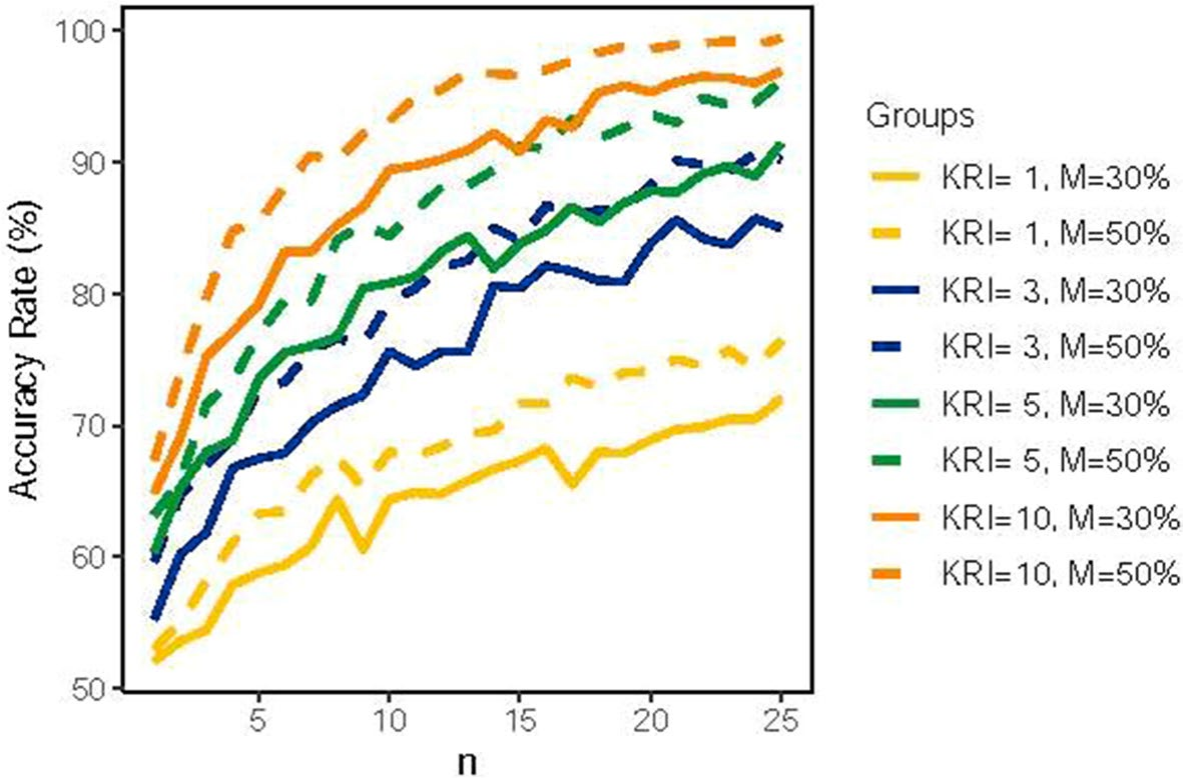
The influence of various numbers of KRIs on the accuracy rate at a single site. *M* represents the percentage of monitoring

Figure 3 shows the influence of different numbers of KRIs on the accuracy rate at a single site. It is evident that the accuracy rate improved and became more stable as more KRIs were used. When using a single KRI, the accuracy rates were mainly within 60% and 70%, regardless of whether 30% or 50% of the monitoring data was collected. Furthermore, when there were 3 KRIs and 30% of the monitoring data was collected, the accuracy rates increased and reached 80% when the total number of subjects was 15.

## Example

In this section, we illustrate how to apply the proposed risk boundaries in practice using data from a real clinical trial (NCT00339183). This is a Phase III multicenter randomised controlled trial (MRCT) with 946 previously treated metastatic colorectal cancer patients across 151 sites. For demonstration purposes, one-third of the sites were randomly selected. The primary objective of the study is to compare the efficacy of panitumumab combined with chemotherapy to the efficacy of chemotherapy alone. The datasets were downloaded from Project Data Sphere. To evaluate the risk level of each site, we selected 4 KRIs: the discontinuation rate and the incidence proportions of dry skin, diarrhoea, and paronychia. The latter three risk levels are listed as frequent AEs for panitumumab in the drug instructions and were counted only once if they occurred multiple times in one subject. We assume that at the same site, the risk level estimates and true incidence proportions of these three AEs are the same. To define the risk levels for these KRIs, we used some specified rules, which were that a site with a discontinuation rate more or less than 30% and 15% of the overall rate or an incidence proportion of AEs more or less than 15% and 10% of the overall incidence proportion were considered as high- and medium-risk, respectively. The overall rate and incidence proportion were estimated by averaging all sites. For clarity, we only identified sites with a high discontinuation rate as high-risk. Although sites with both high and low incidence proportions were considered as high-risk, we only displayed situations where the incidence proportions of AEs were too low, such as sites with unreported AEs. The proposed risk boundaries were determined through the binomial distribution based on the medium- and high-risk levels, using various informative and non-informative priors. The high-risk threshold of the funnel plot was based on a nominal 99.7% confidence interval, which is consistent with Zink et al. [21].

**Fig. 4:**
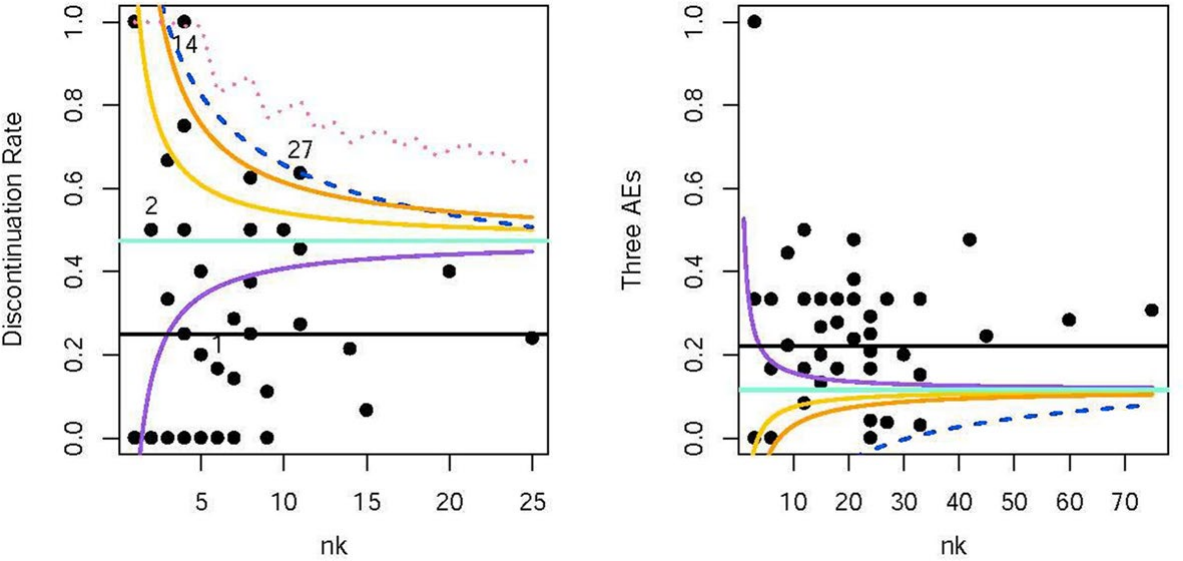
The proposed risk boundaries and the funnel plot in a real clinical trial. The average incidence proportions of the discontinuation and the three AEs are 25.0% and 22.2%, respectively. The high-risk levels of the discontinuation and three AEs are 55.0% and 7.2%. The medium-risk levels of the discontinuation and three AEs are 40.0% and 12.2%. $n_{jk}$ is the number of subjects with the $j^{\text{th}}$ KRI recorded at the $k^{\text{th}}$ site. $n_k=\sum \nolimits _{j=1}^{J}n_{jk}$

We first assessed the risk for the final trial data. In the left panel of Fig. 4, the black line represents the average discontinuation rate across all sites, while the aquamarine line represents the risk boundary calculated using our method with a non-informative prior. Different prior ratios of low and high discontinuation rate sites are represented by the orange, yellow, and purple lines to be 7:3, 6:4, and 4:6, respectively. The threshold of the funnel plot is represented by the dashed blue line. The pink dashed line represents the risk boundary achieved by minimising the average decision error rate without prior information. The final results indicated that sites 14 and 27 were identified as high-risk sites when the prior ratio of low and high discontinuation rate sites was 7:3. This conclusion aligned with that of the funnel plot. As the prior ratio decreased from 7:3 to 4:6, more sites are needed to take some additional monitoring actions, such as on-site monitoring. If this ratio is interpreted as the severity of trial quality that sponsors assume, as described in Non-constant risk rate section, or as the proportion of sites where sponsors prefer not to take additional monitoring actions, then the trend is reasonable. Notably, the purple line indicates that sponsors wanted to take more additional monitoring actions compared to the non-informative prior; this is impossible for the funnel plot to achieve. The pink dashed line has the widest risk boundary since the upper limit of the high-risk estimate for the discontinuation rate was 100%.

The right panel of Fig. 4 illustrates the scenario when three AEs were used to calculate the risk boundaries. Since a lower incidence proportion indicates higher risk, the prior ratios of low and high incidence proportions are respectively represented by the orange, yellow, and purple lines to be 3:7, 4:6, and 6:4, which is the opposite of the discontinuation rate setting. However, the risk assessment results of three AEs can be interpreted in the same way as those of the discontinuation rate. For example, the purple line suggests that there were more sites with low incidence proportions or high risks based on the prior information, or it indicates that sponsors assumed that more sites were at a high-risk level or that they preferred to take more additional monitoring actions. In addition, the threshold of the funnel plot was a little wide, so no site required additional monitoring actions. We can imagine that if we only used one AE as KRI, that is, $n_k$ equaled 25 at most, then only a small segment at the end of the funnel plot is greater than zero, rendering it almost meaningless in terms of risk assessment.

**Fig. 5:**
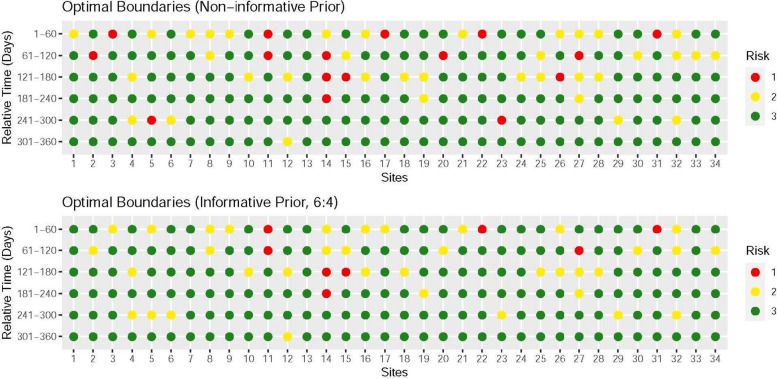
The risk of each site is assessed based on the discontinuation rate within each of the 60-day time window. Only sites with at least one discontinued subject are included in the analysis

Figure 5 shows the risk assessment results based on the discontinuation rate at different stages, divided based on the relative time windows when combined with the traffic light system [15] proposed by TransCelerate in scenarios with a non-informative prior or a prior information ratio of 6:4. Compared to the non-informative prior, the overall risks across the sites decreased when the prior information ratio was 6:4, which aligned with the previous conclusions. For sites 14 and 27, their multiple relative time windows were classified as high or medium risks. By comparing the positions of sites in Fig. 4 with their results in different time windows in Fig. 5, we observed a correspondence between most of them, such as sites 1 and 2.

## Discussion

Through simulations, we observed the relationships between the proposed method and various factors. The risk level estimates and prior information primarily influence whether the proposed boundaries are optimal, while the difference between two neighbouring risk level estimates, the number of subjects at the site, the number of KRIs, and the percentage of the collected monitoring data primarily impact the performance of the proposed risk boundaries. Sponsors have the flexibility to choose either of the two approaches proposed in Establishing risk levels for KRIs along with their corresponding monitoring actions and estimates section for determining risk level estimates, depending on their specific requirements and available information. However, similar to QTLs and thresholds, the accurate definition of risk level estimates is crucial, and it can even be considered a limitation of such methods. Therefore, it is recommended to make reference to historical data or other available criteria as much as possible.

In Simulation section, we demonstrated that the proposed method identified the optimal risk boundaries when the prior information and the true proportions of site risks were consistent. Nevertheless, in practice, the true proportions of high- and low-risk sites are unknown and can vary across different trials, drugs, and even different stages of the trial, as illustrated in the COVID-19 example. The definition of high- and low-risk is highly subjective and often cannot be resolved through a pure technical approach. Therefore, prior information should primarily be applied to adjusting the risk boundaries according to the specific requirements of the trial. The proposed method ensures the identification of the optimal risk boundaries under the given requirements. In Example section, we provide an intuitive explanation for selecting the prior information. The inclusion of prior information allows for flexibility in determining the number of sites to take monitoring actions, which can be adjusted according to the preferences and needs of sponsors. From this perspective, the proposed method offers an adaptable approach to adjusting risk boundaries.

When considering the factors affecting the performance of the proposed method, the difference between risk level estimates is selected based on historical data of similar products, expert advice, or other criteria. Therefore, we mainly focus on discussing the influence of the other three factors. Generally, higher accuracy rates are associated with a larger sample size or monitoring data. This finding is particularly beneficial for large-scale trials or sites, as it allows for cost savings by reducing the frequency of on-site monitoring. Regarding the number of KRIs, when the risk level estimates of KRIs are accurately defined, more KRIs lead to an increased accuracy rate. However, in real trials, accurately defining risk level estimates for numerous KRIs and ensuring their independence can be challenging. Therefore, it is advisable to prioritise the selection of KRIs with a higher level of confidence to effectively incorporate them into the proposed method.

Additionally, there are two issues related to independence that should be considered. First, the independence assumption required by the Poisson distribution should be assessed for events that can occur multiple times. In other words, the occurrence of an event should not be influenced by previous events. For example, if the probability of an AE recurrence significantly decreases after its initial occurrence, the independence assumption may not hold. Second, the independence between the KRIs used for the combination should also be assessed. A common example is when there is a competing risk between two KRIs, which may violate this independence assumption. Another important consideration is the choice between using cumulative data or staged data for risk-based monitoring. Our recommendation is as follows: if a trial or site is not intervened on and the risk rate does not significantly change over time, it is preferable to use the cumulative data. However, in cases involving post-intervention data or situations where the risk rates fluctuate, the staged data, as shown in Example section, can be used for risk-based monitoring.

The proposed risk boundaries provide several distinct advantages. First, the calculation process for the proposed risk boundaries is straightforward and typically does not require extensive programming. Since the proposed method provides analytical solutions, it can be easily used by non-statistical roles in clinical research, such as physicians, project managers, or clinical research associates. In Appendix E, we have developed the SAS macro to calculate risk boundaries based on different distributions. Second, the proposed method is applicable to a wide range of clinical trials, including Phase I to Phase IV, single or multicenter. The proposed risk boundaries can be derived from different KRIs, similar to TransCelerate, or only focus on therapeutic areas or populations, similar to the funnel plot, rather than being limited to a single approach. Although the proposed method can technically be applied at different levels, its practical implementation should adhere to the corresponding rules. For instance, site-level boundaries can be adjusted during the trial, while trial-level boundaries are not recommended for adjustment. We have derived risk boundaries based on four commonly used distributions. The proposed method can also be used for other distributions, such as the Weibull distribution, when applicable. However, it may not have an analytical solution. Third, compared to testing the risks between sites using frequentist methods, the proposed method avoids the issue of multiple testing, thereby streamlining the analysis process. Fourth, the proposed method provides flexibility in incorporating multiple KRIs and assigning different weights to each KRI. This allows for customisation based on the specific risk factors of interest in a given trial.

## Conclusion

In this paper, we propose a Bayesian risk-based monitoring method for assessing the trial quality based on the prespecified KRIs and their risk level estimates. The proposed method distinguishes itself from other approaches through its clear purpose and approach to boundary selection, aiming to identify the optimal risk boundaries that minimise the decision error rate. The proposed risk boundaries are applicable at both the trial- and site-levels, accommodating multiple risk levels and various endpoint types. In conclusion, with the trend shifting from 100% SDV to more targeted monitoring, centralised and risk-based monitoring are playing an increasingly pivotal role in clinical monitoring. Consequently, this direction deserves further study and development.

### Supplementary Information

Supplementary Material 1.

## Data Availability

The data of the case study is available at https://data.projectdatasphere.org/.

## Abbreviations

AE: Adverse event
AESI: Adverse events of special interest
COVID-19: Corona virus disease 2019
EMA: European medicines agency
FDA: Food and drug administration
ICH: International conference on harmonisation of technical requirements & for registration of pharmaceuticals for human use
IDMC: Independent data monitoring committee
KRIs: Key risk indicators
MRCT: Multicenter randomised controlled trial
NDA: New drug application
*Q*1: First quartile
QTLs: Quality tolerance limits
R[MYAMP: D] Research and development
RBM: Risk-based monitoring
SAS: Statistical analysis system
SDV: Source data verification

## Code availability

The SAS codes are available in Appendix E.
