# Supplementary material for: Determining the extent and frequency of on-site monitoring: a bayesian risk-based approach
Source: BMC Med Res Methodol. 2024 Jun 28;24:141. doi: 10.1186/s12874-024-02261-y (PMC11212185; doi:10.1186/s12874-024-02261-y)
Supplement: Supplementary file 1 — Supplementary Material 1. [file 12874_2024_2261_MOESM1_ESM.pdf]

Appendix A Derivation for the optimal risk boundaries

For the Poisson process,

$$
\begin{aligned}
\Pr(\text{Error}) &= \sum_{g=1}^{G} \Pr(\mathrm{H}_{gk}) \Pr(\overline{S}_g | \, \mathrm{H}_{gk}) \\
&= \sum_{g=1}^{G-1} \left\{ \Pr(\mathrm{H}_{gk}) \, \mathrm{P}(\Theta_{gk}; \lambda_g t_k) - \Pr(\mathrm{H}_{(g+1)k}) \, \mathrm{P}(\Theta_{gk}; \lambda_{g+1} t_k) \right\} + \sum_{g=2}^{G} \Pr(\mathrm{H}_{gk}),
\end{aligned}
$$

where $\lambda_1 t_k > \lambda_2 t_k > \ldots > \lambda_G t_k$. H_{gk}:$\Lambda_k t_k = \lambda_g t_k$. We define

$$
\begin{aligned}
\varphi_g &\triangleq \Pr\left(\mathrm{H}_{gk}\right) \mathrm{P}\left(\Theta_{gk}; \lambda_g t_k\right) \; - \Pr\left(\mathrm{H}_{(g+1)k}\right) \; \mathrm{P}\left(\Theta_{gk}; \lambda_{g+1} t_k\right) \\
&= \Pr\left(\mathrm{H}_{gk}\right) \sum_{\tau_k=0}^{\lfloor \Theta_{gk} \rfloor} e^{-\lambda_g t_k} \frac{(\lambda_g t_k)^{\tau_k}}{\tau_k!} - \Pr\left(\mathrm{H}_{(g+1)k}\right) \sum_{\tau_k=0}^{\lfloor \Theta_{gk} \rfloor} e^{-\lambda_{g+1} t_k} \frac{(\lambda_{g+1} t_k)^{\tau_k}}{\tau_k!} \\
&= \sum_{\tau_k=0}^{\lfloor \Theta_{gk} \rfloor} \{ \Pr\left(\mathrm{H}_{gk}\right) \, e^{-\lambda_g t_k} \frac{(\lambda_g t_k)^{\tau_k}}{\tau_k!} - \Pr\left(\mathrm{H}_{(g+1)k}\right) \, e^{-\lambda_{g+1} t_k} \frac{(\lambda_{g+1} t_k)^{\tau_k}}{\tau_k!} \} \\
&= \sum_{\tau=0}^{\lfloor \Theta_{gk} \rfloor} [\Pr\left(\mathrm{H}_{(g+1)k}\right) \, e^{-\lambda_{g+1} t_k} \frac{(\lambda_{g+1} t_k)^{\tau_k}}{\tau_k!} \{ \frac{\Pr\left(\mathrm{H}_{gk}\right) \, e^{-\lambda_g t_k} (\lambda_g t_k)^{\tau_k}}{\Pr\left(\mathrm{H}_{(g+1)k}\right) \, e^{-\lambda_{g+1} t_k} (\lambda_{g+1} t_k)^{\tau_k}} - 1 \}],
\end{aligned}
$$

where $\lambda_g t_k = \sum_{j=1}^{J} w_j \lambda_{jg} t_{jk}$ and $J \geq 1$. Since $\lambda_{g+1} t_k < \lambda_g t_k$, $(\frac{\lambda_g t_k}{\lambda_{g+1} t_k})^{\tau_k}$ increases with τ_k monotonically. Thus φ_g is minimised when $\frac{\Pr(\mathrm{H}_{gk}) \; e^{-\lambda_g t_k} (\lambda_g t_k)^{\Theta_{gk}}}{\Pr\left(\mathrm{H}_{(g+1)k}\right) \; e^{-\lambda_{g+1} t_k} (\lambda_{g+1} t_k)^{\Theta_{gk}}} \leq 1$. We obtain

$$
\Theta_{gk} = \frac{\ln \left(\frac{\Pr\left(\mathrm{H}_{(g+1)k}\right)}{\Pr(\mathrm{H}_{gk})} \right)}{\ln \lambda_g t_k - \ln \lambda_{g+1} t_k} + \frac{\lambda_g t_k - \lambda_{g+1} t_k}{\ln \lambda_g t_k - \ln \lambda_{g+1} t_k}.
$$

When we use the Poisson distribution, the risk boundary is

$$\theta_{gk} = \frac{\ln\left(\frac{\Pr\left(\mathrm{H}_{(g+1)k}\right)}{\Pr(\mathrm{H}_{gk})}\right)}{\ln\lambda_g - \ln\lambda_{g+1}} + \frac{\lambda_g - \lambda_{g+1}}{\ln\lambda_g - \ln\lambda_{g+1}},$$

where H_{gk}:$\Lambda_k = \lambda_g$. Using the same approach, we obtain the risk boundary for the exponential distribution

$$e_{jgk} = \frac{\ln\left(\frac{\Pr\left(\mathrm{H}_{j(g+1)k}\right)}{\Pr(\mathrm{H}_{jgk})}\right)}{\lambda_{jg} - \lambda_{j(g+1)}} + \frac{\ln\lambda_{jg} - \ln\lambda_{j(g+1)}}{\lambda_{jg} - \lambda_{j(g+1)}},$$

where assuming that the jth KRI results at the gth risk level follow $\mathrm{Exp}(\lambda_{jg})$. H_{jgk}:$\Lambda_{jk} = \lambda_{jg}$. When $J = 1$ and non-informative prior, $e_{jgk} = \frac{1}{\theta_{gk}}$.

Similarly, the risk boundary for the normal distribution

$$\delta_{gk} = \frac{\ln\left(\frac{\Pr\left(\mathrm{H}_{(g+1)k}\right)}{\Pr(\mathrm{H}_{gk})}\right)\sigma^2}{(\mu_g - \mu_{g+1})} + \frac{(\mu_g + \mu_{g+1})}{2},$$

where assuming that the KRIs results at the gth risk level follow $\mathcal{N}(\mu_g, \sigma_g^2)$ and $\mu_g = \sum_{j=1}^{J} w_j \mu_{jg}$, $\mu_1 > \mu_2 > \cdots > \mu_g$, $\sigma_1^2 = \sigma_2^2 = \cdots = \sigma_G^2 = \sum_{j=1}^{J} \sigma_{jg}^2 = \sigma^2$, and $J \geq 1$. H_{gk}:$M_k = \mu_g$.

For the binomial distribution

$$\pi_{gk} = \frac{n_k^{-1}\ln\left(\frac{\Pr\left(\mathrm{H}_{(g+1)k}\right)}{\Pr(\mathrm{H}_{gk})}\right)}{\ln\left(\frac{p_g(1-p_{g+1})}{p_{g+1}(1-p_g)}\right)} + \frac{\ln\left(\frac{1-p_{g+1}}{1-p_g}\right)}{\ln\left(\frac{p_g(1-p_{g+1})}{p_{g+1}(1-p_g)}\right)},$$

where $n_k = \sum_{j=1}^{J} n_{jk}$, $p_g = \frac{\sum_{j=1}^{J} w_j p_{jg}}{J}$, $p_1 > p_2 > \cdots > p_g$, and $J \geq 1$. n_{jk} is the number of subjects having the observed result of the jth KRI at the kth site. Assuming

that the KRIs results at the gth risk level follow $\text{Bin}(p_g, n_k)$. H_{gk}:$P_k = p_g$. Note when $p_g + p_{g+1} = 1$ and $\Pr(\text{H}_{gk}) = \Pr\left(\text{H}_{(g+1)k}\right)$, we have

$$\pi_{gk} = \frac{\ln\left(\frac{p_g}{1-p_g}\right)}{\ln\left(\frac{p_g p_g}{(1-p_g)(1-p_g)}\right)} = \frac{1}{2}.$$

Appendix B Derivation for minimising the average decision error rate

For the Poisson distribution

$$\begin{aligned}\Pr(\text{Error}) &= \sum_{g=2}^{G} \Pr(\text{H}_{gk}) \int_{\lambda_g}^{\lambda_{g-1}} f(\Lambda_k|\text{H}_{gk}) \Pr(\overline{S}_{g-1}|\text{H}_{gk}) d\Lambda_k \\ &= \sum_{g=2}^{G-1} \sum_{\tau=0}^{\lfloor \theta_{(g-1)k}-1 \rfloor} \left[f(\tau)\{\Pr(\text{H}_{gk}|\tau) - \Pr(\text{H}_{(g+1)k}|\tau)\}\right] + \sum_{g=2}^{G} \Pr(\text{H}_{gk}).\end{aligned}$$

Due to

$$\Pr(\text{H}_{gk}|\tau) = \frac{\Pr(\text{H}_{gk}) \int_{\lambda_g}^{\lambda_{g-1}} f(\Lambda_k|\text{H}_{gk}) f(\tau|\Lambda_k, \text{H}_{gk}) d\Lambda_k}{f(\tau)}$$

and

$$\begin{aligned}\int_{\lambda_g}^{\lambda_{g-1}} f(\Lambda_k|\text{H}_{gk})\, f(\tau|\Lambda_k, \text{H}_{gk})\, d\Lambda_k &= \frac{\int_{\lambda_g}^{\lambda_{g-1}} \frac{e^{-\Lambda_k}\Lambda_k^{\tau}}{\tau!} d\Lambda_k}{\lambda_{g-1} - \lambda_g} \\ &= \frac{\int_0^{\lambda_{g-1}} \frac{e^{-\Lambda_k}\Lambda_k^{\tau}}{\tau!} d\Lambda_k - \int_0^{\lambda_g} \frac{e^{-\Lambda_k}\Lambda_k^{\tau}}{\tau!} d\Lambda_k}{\lambda_{g-1} - \lambda_g}\end{aligned}$$

and

$$\int_0^{\lambda_g} \frac{e^{-\Lambda_k}\Lambda_k^{\tau}}{\tau!} d\Lambda_k = \frac{1}{\tau!} \int_0^{\lambda_g} e^{-\Lambda_k}\Lambda_k^{\tau} d\Lambda_k$$

$$
\begin{aligned}
&= \frac{1}{\tau!}\gamma\left(\tau+1,\ \lambda_g\right) \\
&= \frac{1}{\tau!}\ \mathrm{Pois}\left(\tau+1;\ \lambda_g\right)\mathit{\Gamma}\left(\tau+1\right) \\
&= \mathrm{Pois}\left(\tau+1;\ \lambda_g\right),
\end{aligned}
$$

where $\mathit{\Gamma}\left(\tau+1\right) = \tau!$ is the Gamma function. $\gamma(\tau+1,\ \lambda_g)$ is the lower incomplete gamma function. $\mathrm{Pois}\left(\tau+1;\ \lambda_g\right)$ is the CDF of the Poisson distribution. H_{gk}:$\lambda_g \leq \Lambda_k < \lambda_{g-1}$.

Then we define

$$
\begin{aligned}
\rho_g &\triangleq \sum_{\tau=0}^{\lfloor\theta_{(g-1)k}\rfloor-1} f\left(\tau\right)\left\{\Pr\left(\mathrm{H}_{gk}|\tau\right) - \Pr\left(\mathrm{H}_{(g+1)k}|\tau\right)\right\} \\
&= \sum_{\tau=0}^{\lfloor\theta_{(g-1)k}\rfloor-1} f\left(\tau\right)\Pr\left(\mathrm{H}_{(g+1)k}|\tau\right)\left\{\frac{\Pr\left(\mathrm{H}_{gk}|\tau\right)}{\Pr\left(\mathrm{H}_{(g+1)k}|\tau\right)} - 1\right\},
\end{aligned}
$$

where

$$
\frac{\Pr\left(\mathrm{H}_{gk}|\tau\right)}{\Pr\left(\mathrm{H}_{(g+1)k}|\tau\right)} = \frac{\Pr\left(\mathrm{H}_{gk}\right)\left(\lambda_g-\lambda_{g+1}\right)}{\Pr\left(\mathrm{H}_{(g+1)k}\right)\left(\lambda_{g-1}-\lambda_g\right)}\frac{\left\{\mathrm{Pois}\left(\tau+1;\ \lambda_{g-1}\right)-\mathrm{Pois}\left(\tau+1;\ \lambda_g\right)\right\}}{\left\{\mathrm{Pois}\left(\tau+1;\ \lambda_g\right)-\mathrm{Pois}\left(\tau+1;\ \lambda_{g+1}\right)\right\}}.
$$

Next, we need to prove the monotonicity. Since $\mathrm{Pois}\left(\tau+1;\lambda_g\right) = e^{-\lambda_g}\sum_{\tau_k=0}^{\tau}\frac{\lambda_g^{\tau_k}}{\tau_k!}$. Then

$$
\begin{aligned}
\frac{\mathrm{Pois}\left(\tau+1;\ \lambda_{g-1}\right)-\mathrm{Pois}\left(\tau+1;\ \lambda_g\right)}{\mathrm{Pois}\left(\tau+1;\ \lambda_g\right)-\mathrm{Pois}\left(\tau+1;\ \lambda_{g+1}\right)} &= \frac{e^{-\lambda_{g-1}}\sum_{\tau_k=0}^{\tau}\lambda_{g-1}^{\tau_k} - e^{-\lambda_g}\sum_{\tau_k=0}^{\tau}\lambda_g^{\tau_k}}{e^{-\lambda_g}\sum_{\tau_k=0}^{\tau}\lambda_g^{\tau_k} - e^{-\lambda_{g+1}}\sum_{\tau_k=0}^{\tau}\lambda_{g+1}^{\tau_k}} \\
&= \frac{\frac{e^{-\lambda_{g-1}}}{e^{-\lambda_g}}\sum_{\tau_k=0}^{\tau}\left(\frac{\lambda_{g-1}}{\lambda_g}\right)^{\tau_k} - 1}{1 - \frac{e^{-\lambda_{g+1}}}{e^{-\lambda_g}}\sum_{\tau_k=0}^{\tau}\left(\frac{\lambda_{g+1}}{\lambda_g}\right)^{\tau_k}}.
\end{aligned}
$$

Now we define τ+1, which is given by

$$\frac{\frac{e^{-\lambda_{g-1}}}{e^{-\lambda_g}}\{(\frac{\lambda_{g-1}}{\lambda_g})^{\tau+1}+\sum_{\tau_k=0}^{\tau}(\frac{\lambda_{g-1}}{\lambda_g})^{\tau_k}\}-1}{1-\frac{e^{-\lambda_{g+1}}}{e^{-\lambda_g}}\{(\frac{\lambda_{g+1}}{\lambda_g})^{\tau+1}+\sum_{\tau_k=0}^{\tau}(\frac{\lambda_{g+1}}{\lambda_g})^{\tau_k}\}}.$$

Because of $\lambda_{g-1} > \lambda_g > \lambda_{g+1} > 0$, we have

$$\frac{\frac{e^{-\lambda_{g-1}}}{e^{-\lambda_g}}\sum_{\tau_k=0}^{\tau}(\frac{\lambda_{g-1}}{\lambda_g})^{\tau_k}-1}{1-\frac{e^{-\lambda_{g+1}}}{e^{-\lambda_g}}\sum_{\tau_k=0}^{\tau}(\frac{\lambda_{g+1}}{\lambda_g})^{\tau_k}} < \frac{\frac{e^{-\lambda_{g-1}}}{e^{-\lambda_g}}\{(\frac{\lambda_{g-1}}{\lambda_g})^{\tau+1}+\sum_{\tau_k=0}^{\tau}(\frac{\lambda_{g-1}}{\lambda_g})^{\tau_k}\}-1}{1-\frac{e^{-\lambda_{g+1}}}{e^{-\lambda_g}}\{(\frac{\lambda_{g+1}}{\lambda_g})^{\tau+1}+\sum_{\tau_k=0}^{\tau}(\frac{\lambda_{g+1}}{\lambda_g})^{\tau_k}\}},$$

which increases with τ monotonically. Similar derivation applies to the Poisson process.

Now we provide the key steps to calculate the risk boundaries for other distributions that minimise the average decision error rate.

For the Exponential distribution based on the Jth KRI

$$\begin{aligned} f(t\,|\,\Lambda_{jk},\mathrm{H}_{jgk}) &= \int_{\lambda_{jg}}^{\lambda_{j(g-1)}} \Lambda_{jk}e^{-\Lambda_{jk}t}d\Lambda_{jk} \\ &= \int_{0}^{\lambda_{j(g-1)}} \Lambda_{jk}e^{-\Lambda_{jk}t}d\Lambda_{jk} \;-\int_{0}^{\lambda_{jg}} \Lambda_{jk}e^{-\Lambda_{jk}t}d\Lambda_{jk} \end{aligned}$$

and

$$\int_{0}^{\lambda_{jg}} \Lambda_{jk}e^{-\Lambda_{jk}t}d\Lambda_{jk} \;=\; \frac{\exp(t;\lambda_{jg})}{t^2}-\frac{\lambda_{jg}e^{-\lambda_{jg}t}}{t},$$

where $\exp(t;\lambda_{jg})$ is the CDF of the exponential distribution. H_{jgk}:$\lambda_{jg} \le \Lambda_{jk} < \lambda_{j(g-1)}$.

For the binomial distribution:

$$\begin{aligned} f(y\,|\,P_k,\mathrm{H}_{gk}) &= \int_{p_g}^{p_{g-1}} \binom{n_k}{y} P_k^y(1-P_k)^{n_k-y}dP_k \\ &= \int_{0}^{p_{g-1}} \binom{n_k}{y} P_k^y(1-P_k)^{n_k-y}dP_k \;-\int_{0}^{p_g} \binom{n_k}{y} P_k^y(1-P_k)^{n_k-y}dP_k \end{aligned}$$

and

$$\int_0^{p_g} \binom{n_k}{y} P_k^y (1-P_k)^{n_k-y} dP_k = \binom{n_k}{y} B_x\left(p_g,\ y+1, n_k-y+1\right)$$

$$= \binom{n_k}{y} \operatorname{Beta}\left(p_g;\ y+1, n_k-y+1\right) B(p_g, y+1, n_k-y+1)$$

$$= \operatorname{Beta}\left(p_g;\ y+1, n_k-y+1\right) \frac{1}{(n_k+1)},$$

where $B_x\left(p_g,\ y+1, n_k-y+1\right)$ is the incomplete beta function. $B(p_g, y+1, n_k-y+1)$ is the beta function. $\operatorname{Beta}\left(p_g;\ y+1, n_k-y+1\right)$ is the CDF of the beta distribution. H_{gk}:$p_g \leq P_k < p_{g-1}$.

For the normal distribution:

$$f\left(x \mid M_k, \mathrm{H}_{gk}\right) = \int_{\mu_g}^{\mu_{g-1}} \frac{1}{\sqrt{2\pi}\sigma_g} e^{\left(-\frac{(x-M_k)^2}{2\sigma_g^2}\right)} dM_k$$

$$= \int_0^{\mu_{g-1}} \frac{1}{\sqrt{2\pi}\sigma_g} e^{\left(-\frac{(x-M_k)^2}{2\sigma_g^2}\right)} dM_k - \int_0^{\mu_g} \frac{1}{\sqrt{2\pi}\sigma_g} e^{\left(-\frac{(x-M_k)^2}{2\sigma_g^2}\right)} dM_k$$

and

$$\int_0^{\mu_g} \frac{1}{\sqrt{2\pi}\sigma_g} e^{\left(-\frac{(x-M_k)^2}{2\sigma_g^2}\right)} dM_k = \frac{1}{\sqrt{2\pi}\sigma_g} \int_0^{\mu_g} e^{\left(-\frac{(x-M_k)^2}{2\sigma_g^2}\right)} dM_k$$

$$= \frac{1}{2} \operatorname{erf}\left(\frac{x-\mu_g}{\sqrt{2}\sigma_g}\right)$$

$$= \mathcal{N}\left(x;\ \mu_g, \sigma_g^2\ \right) - \frac{1}{2},$$

where $\operatorname{erf}\left(\frac{x-\mu_g}{\sqrt{2}\sigma_g}\right)$ is the error function. $\mathcal{N}\left(x;\ \mu_g, \sigma_g^2\ \right)$ is the CDF of the normal distribution. H_{gk}:$\mu_g \leq M_k < \mu_{g-1}$.

Appendix C Comparing the risk boundaries base on binomial and converted Poisson process

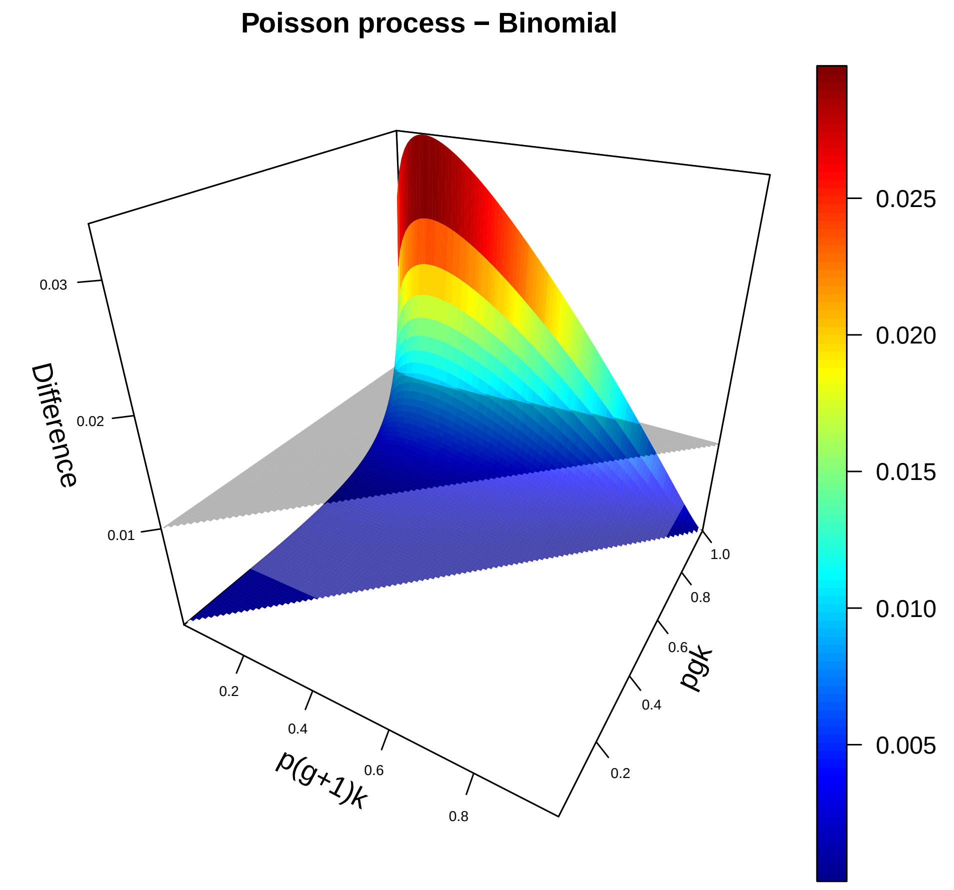

Fig.S1 The difference between the risk boundaries derived based on the binomial distribution and converted from the Poisson process when the KRI can only occur once

Appendix D The performances of the proposed risk boundaries when a informative prior is used

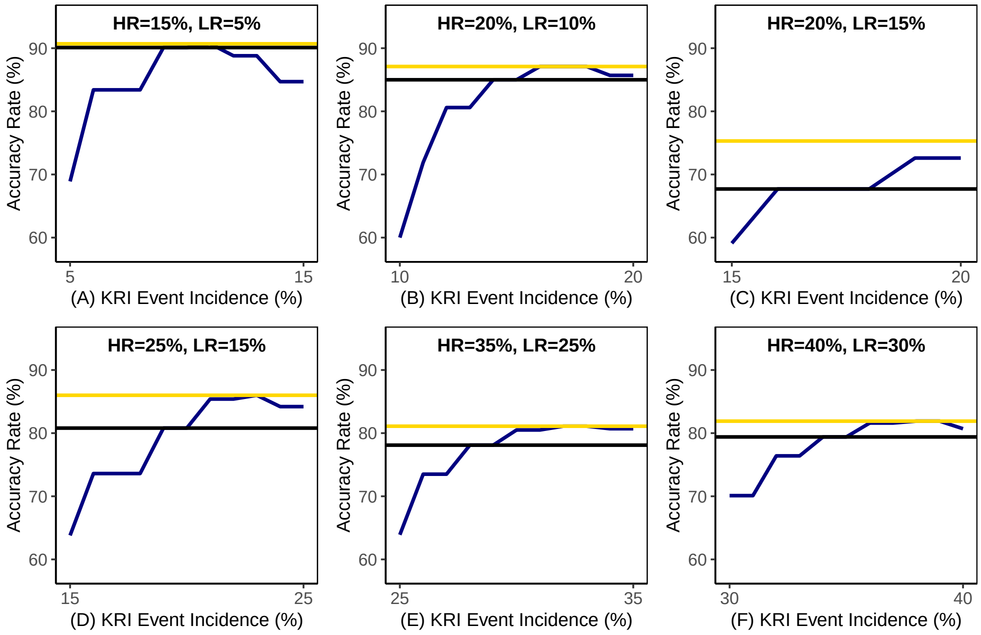

Fig.S2 The comparison of the performances between the proposed risk boundaries and all other risk boundaries when a prior information is used

Appendix E SAS code for calculating risk boundaries.

Poisson Process

```
%macro PP(pr1, pr2, lambda1, lambda2, tk);
data boundary;
pr1=&pr1;
pr2=&pr2;
lambda1=&lambda1;
lambda2=&lambda2;
tk=&tk;
boundary=log(&pr2/&pr1)/(log(&lambda1*&tk)-log(&lambda2*&tk))+(&lambda1*&tk-
&lambda2*&tk)/(log(&lambda1*&tk)-log(&lambda2*&tk));
run;

proc report data=boundary headline headskip nowindows missing split="*";
title "Risk Boundary based on Poisson Process";
column pr1 pr2 lambda1 lambda2 tk boundary;
define pr1 / width=14 center "Prior Probability for High-Risk Level";
define pr2 / width=14 center "Prior Probability for Low-Risk Level";
define lambda1 / width=14 center "Incidence Rate for High-Risk Level";
define lambda2 / width=14 center "Incidence Rate for Low-Risk Level";
define tk / width=14 center "Follow-Up Time";
define boundary / width=14 center "Risk Boundary";
run;
%mend;
```

Poisson Distribution

```
%macro PD(pr1, pr2, lambda1, lambda2);
data boundary;
pr1=&pr1;
pr2=&pr2;
lambda1=&lambda1;
lambda2=&lambda2;
boundary=log(&pr2/&pr1)/(log(&lambda1)-log(&lambda2))+(&lambda1-
&lambda2)/(log(&lambda1)-log(&lambda2));
run;

proc report data=boundary headline headskip nowindows missing split="*";
title "Risk Boundary based on Poisson Distribution";
column pr1 pr2 lambda1 lambda2 boundary;
define pr1 / width=14 center "Prior Probability for High-Risk Level";
define pr2 / width=14 center "Prior Probability for Low-Risk Level";
define lambda1 / width=14 center "Incidence Rate for High-Risk Level";
define lambda2 / width=14 center "Incidence Rate for Low-Risk Level";
define boundary / width=14 center "Risk Boundary";
run;
%mend;
```

Binomial Distribution

```
%macro BD(pr1, pr2, p1, p2, nk);
data boundary;
pr1=&pr1;
pr2=&pr2;
```

```
prop1=&p1;
prop2=&p2;
nk=&nk;
boundary=log(&pr2/&pr1)/(&nk*log(&p1*(1-&p2)/(&p2*(1-&p1))))+log((1-
&p2)/(1-&p1))/log(&p1*(1-&p2)/(&p2*(1-&p1)));
run;

proc report data=boundary headline headskip nowindows missing split="*";
title "Risk Boundary based on Binomial Distribution";
column pr1 pr2 prop1 prop2 nk boundary;
define pr1 / width=14 center "Prior Probability for High-Risk Level";
define pr2 / width=14 center "Prior Probability for Low-Risk Level";
define prop1 / width=14 center "Incidence Proportion for High-Risk Level";
define prop2 / width=14 center "Incidence Proportion for Low-Risk Level";
define nk / width=14 center "Number of Subjects";
define boundary / width=14 center "Risk Boundary";
run;
%mend;
```

Normal Distribution

```
%macro ND(pr1, pr2, mu1, mu2, sigma, nk);
data boundary;
pr1=&pr1;
pr2=&pr2;
mu1=&mu1;
mu2=&mu2;
sigma=&sigma;
```

```
boundary=log(&pr2/&pr1)*&sigma*&sigma/(&mu1-&mu2)+(&mu1+&mu2)/2;
run;

proc report data=boundary headline headskip nowindows missing split=”*”;
title ”Risk Boundary based on Normal Distribution”;
column pr1 pr2 mu1 mu2 sigma boundary;
define pr1 / width=14 center ”Prior Probability for High-Risk Level”;
define pr2 / width=14 center ”Prior Probability for Low-Risk Level”;
define mu1 / width=14 center ”Mean for High-Risk Level”;
define mu2 / width=14 center ”Mean for Low-Risk Level”;
define sigma / width=14 center ”Standard Deviation”;
define boundary / width=14 center ”Risk Boundary”;
run;
%mend;
```

Exponential Distribution

```
%macro ED(pr1, pr2, lambda1, lambda2);
data boundary;
pr1=&pr1;
pr2=&pr2;
lambda1=&lambda1;
lambda2=&lambda2;
boundary=log(&pr2/&pr1)/(&lambda1-&lambda2)+(log(&lambda1)-
log(&lambda2))/(&lambda1-&lambda2);
run;

proc report data=boundary headline headskip nowindows missing split=”*”;
```

```
title "Risk Boundary based on Exponential Distribution";
column pr1 pr2 lambda1 lambda2 boundary;
define pr1 / width=14 center "Prior Probability for High-Risk Level";
define pr2 / width=14 center "Prior Probability for Low-Risk Level";
define lambda1 / width=14 center "Incidence Rate for High-Risk Level";
define lambda2 / width=14 center "Incidence Rate for Low-Risk Level";
define boundary / width=14 center "Risk Boundary";
run;
%mend;
```

Appendix F Summary of optimal risk boundaries by distribution

Table S1 The application summary of the optimal risk boundaries for various distributions

Distribution	Optimal Risk Boundaries	Comparison Targets	Assessment 1st*	Assessment 2nd*	KRI Single	KRI Combined
Poisson Process	$\frac{\ln\left(\frac{\Pr\left(\mathrm{H}_{(g+1)k}\right)}{\Pr\left(\mathrm{H}_{gk}\right)}\right)}{\ln\lambda_g t_k - \ln\lambda_{g+1} t_k} + \frac{\lambda_g t_k - \lambda_{g+1} t_k}{\ln\lambda_g t_k - \ln\lambda_{g+1} t_k}$	Average total number of events of all subjects at the kth site or trial until time t	✓	✓	✓	✓
Poisson Distribution	$\frac{\ln\left(\frac{\Pr\left(\mathrm{H}_{(g+1)k}\right)}{\Pr\left(\mathrm{H}_{gk}\right)}\right)}{\ln\lambda_g - \ln\lambda_{g+1}} + \frac{\lambda_g - \lambda_{g+1}}{\ln\lambda_g - \ln\lambda_{g+1}}$	Average number of events of all subjects at the kth site or trial in a unit time	✓		✓	✓
Binomial	$\frac{n_k^{-1}\ln\left(\frac{\Pr\left(\mathrm{H}_{(g+1)k}\right)}{\Pr\left(\mathrm{H}_{gk}\right)}\right)}{\ln\left(\frac{p_g(1-p_{g+1})}{p_{g+1}(1-p_g)}\right)} + \frac{\ln\left(\frac{1-p_{g+1}}{1-p_g}\right)}{\ln\left(\frac{p_g(1-p_{g+1})}{p_{g+1}(1-p_g)}\right)}$	Incidence proportion at the kth site or trial	✓	✓	✓	✓
Exponential	$\frac{\ln\left(\frac{\Pr\left(\mathrm{H}_{j(g+1)k}\right)}{\Pr\left(\mathrm{H}_{jgk}\right)}\right)}{\lambda_{jg} - \lambda_{j(g+1)}} + \frac{\ln\lambda_{jg} - \ln\lambda_{j(g+1)}}{\lambda_{jg} - \lambda_{j(g+1)}}$	Quantile survival time at the kth site or trial	✓		✓	
Normal	$\frac{\ln\left(\frac{\Pr\left(\mathrm{H}_{(g+1)k}\right)}{\Pr\left(\mathrm{H}_{gk}\right)}\right)\sigma^2}{(\mu_g - \mu_{g+1})} + \frac{(\mu_g + \mu_{g+1})}{2}$	Average observed value of all subjects at the kth site or trial	✓		✓	✓

1st* represents the risk can be assessed through specified rules as mentioned in Section 2.1.
2nd* represents the risk can be assessed based on different monitoring time as mentioned in Sections 2.2 and 2.4.
